# NETs - as predictors and targets of supportive therapy for cancer treatment

**DOI:** 10.3389/fimmu.2025.1666261

**Published:** 2025-09-12

**Authors:** Maria-Laura Morawiec, Robert Kubina, Ewa Jabłońska, Wioletta Ratajczak-Wrona, Sebastian Stępień, Maciej Gołębski, Aleksandra Mielczarek-Palacz

**Affiliations:** ^1^ Department of Immunology and Serology, Faculty of Pharmaceutical Sciences in Sosnowiec, Medical University of Silesia in Katowice, Katowice, Poland; ^2^ Silesia LabMed: Centre for Research and Implementation, Medical University of Silesia in Katowice, Katowice, Poland; ^3^ Department of Pathology, Faculty of Pharmaceutical Sciences in Sosnowiec, Medical University of Silesia in Katowice, Katowice, Poland; ^4^ Department of Immunology, Medical University of Bialystok, Białystok, Poland

**Keywords:** NETs, therapy, cancer, chemoresistance, DNase 1

## Abstract

NETs are network-like structures consisting mainly of DNA and various proteins released by neutrophils physiologically in response to pathogens. Moreover, according to recent reports, NETs also play an important role in carcinogenesis. They are involved in all stages of carcinogenesis, assist in the process of metastasis, and their presence has been linked to higher mortality and poorer prognosis in numerous cancer types. This review focuses on anti-cancer treatments related to disintegration of existing NETs, inhibition of their formation and regulation of their formation. Cases in which the presence of NETs was associated with anti-cancer activity and the association of NETs with complications co-occurring with cancer or related to cancer treatment was presented. This paper also presents mechanisms of NETs inhibition, predicting the efficacy or resistance of anti-cancer therapy associated with NETs.

## 1 Introduction

**Figure 1:**
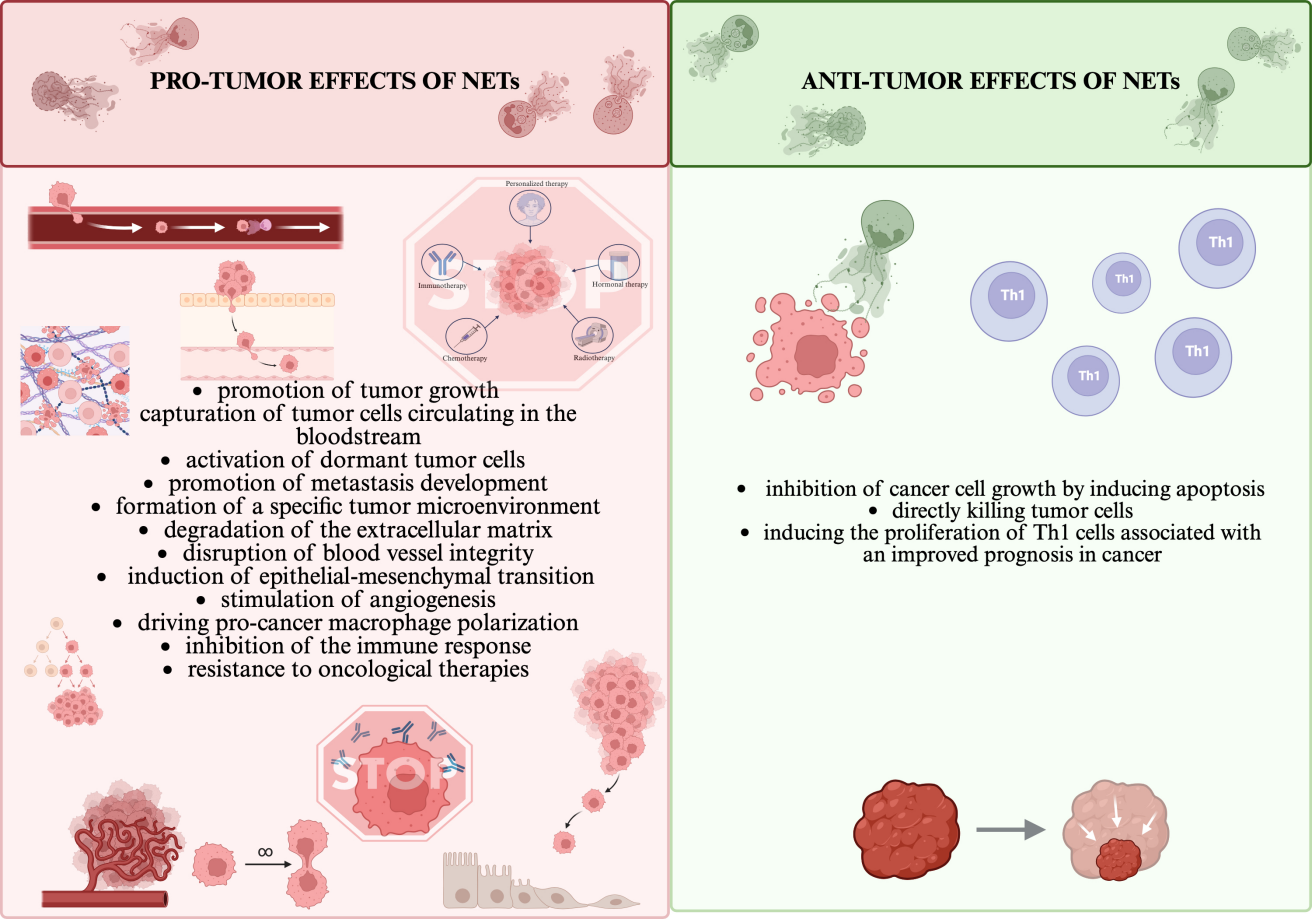
NETs’ dual roles in pro/anti-tumor effects (1, 6–9). All the figures presented in the paper were created in https://BioRender.com.

Neutrophil extracellular traps (NETs) are formed by networks composed of deoxyribonucleic acid (DNA) and protein components, including neutrophil elastase (NE), histones, proteases, myeloperoxidase (MPO), lactoferrin, defensin, lysozyme C, cathelicidin, calprotectin, cathepsin G (CTSG) and matrix metalloproteinase-9 (MMP-9) (1). The main physiological role of NETs is to capture various pathogens, while their presence and excessive production have also been detected in numerous cancers (2). NETs act in a dichotomous manner, their effects can be both pro- and anti-tumor depending on the state of the immune system or the tumor microenvironment (TME) (3, 4). In contrast, it has been shown that patients with NETs involved in the tumor process showed a less favourable prognosis of the disease and a higher mortality rate (5, 6). A summary and comparison of the bidirectional role of NETs is presented in **Figure 1**. It should be emphasized that the function of NETs varies depending on the type of cancer.

The interaction of tumor cells with NETs plays an important role in evading the immune response (10). NETs present in TME have the ability to form a physical barrier that prevents immune effector cells: NK cells (Natural Killer), CD8+ T lymphocytes, cytotoxic T lymphocytes (CTL) from coming into contact with tumor cells, thereby mitigating their anti-tumor effects, primarily the elimination of tumor cells (10, 11). NETs can induce Th1 cell proliferation, which is associated with improved cancer prognosis, but at the same time they can drive macrophage polarization and then cooperatively promote tumor cell invasion and metastasis (7). NETs have also been shown to promote cancer metastasis by trapping circulating tumor cells (CTCs), which, when caught in the network, are protected from degradation and translocated to sites of potential metastasis (10, 12). In addition, NETs have the ability to degrade the extracellular matrix, disrupt blood vessel integrity and activate dormant tumor cells (13). NETs are also associated with complications associated with cancer, which include chronic inflammation, impairment of peripheral vascular and organ function, primarily the kidney, and thrombosis (14).

In cancer patients, under the influence of chemotactic factors, not only neutrophils but also granulocytic myeloid-derived suppressor cells (MDSCs) produce NETs (11). Various types of cancer cells and TME can induce the formation of NETs (15–17). Also, the stress related to surgery, often performed as part of anti-neoplastic treatment, can stimulate the formation of NETs, accelerating the development of the disease (18). NETs formation is also influenced by the anticancer treatment itself, primarily chemotherapy, radiotherapy and immunotherapy (19–21). Disintegration of existing NETs, inhibition of their formation or regulation of their formation therefore represents a potential therapeutic target for both primary and metastatic cancers (10, 22, 23).

**Figure 2:**
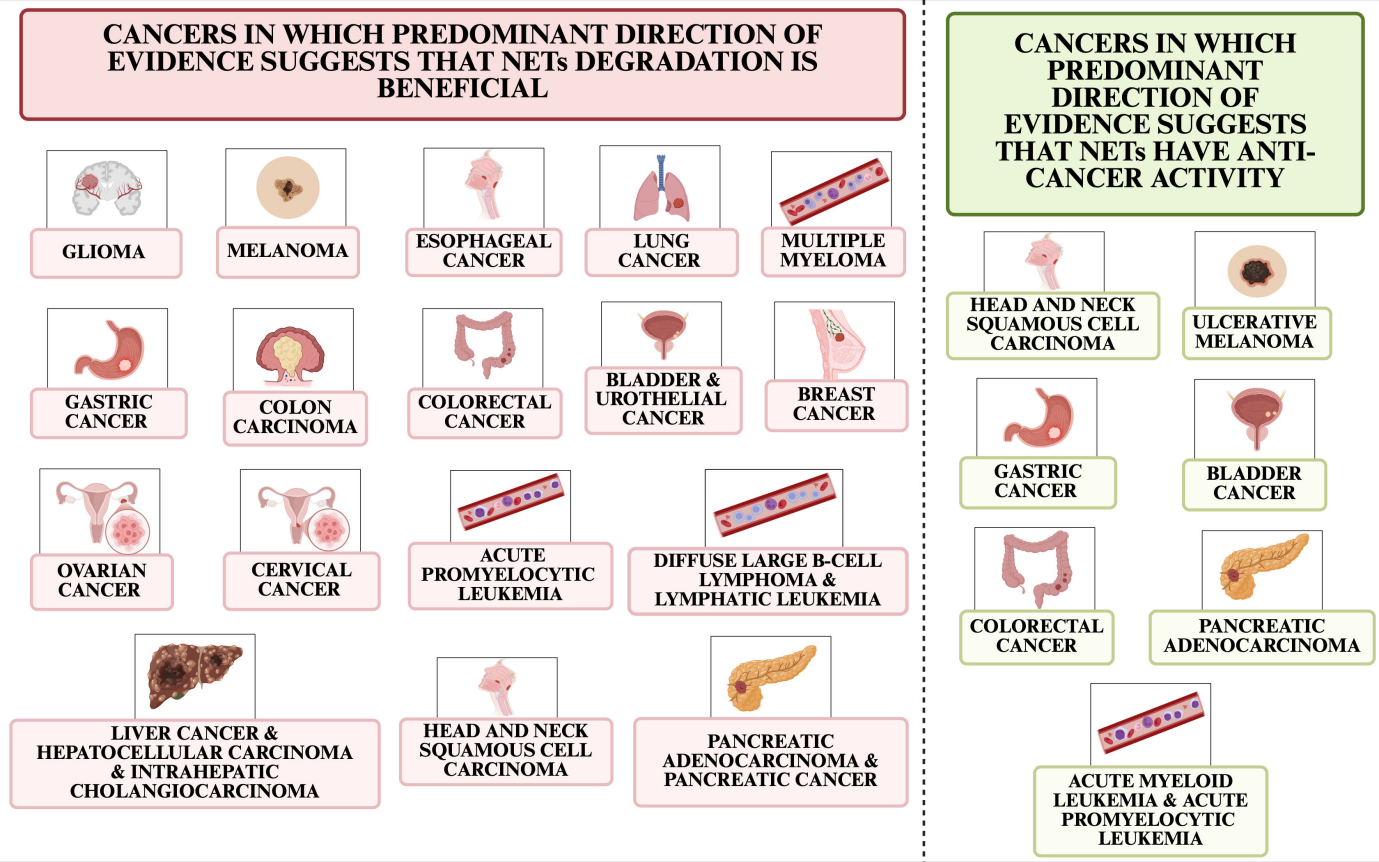
Cancer types in which predominant direction of evidence suggests that NETs degradation is beneficial and those in which evidence suggests NETs have anti-cancer effects.

This paper presents the mechanisms of NETs inhibition, the prediction of efficacy or resistance of anticancer therapy associated with NETs, the mechanisms of anti-cancer therapy associated with blocking or exploiting NETs, and current clinical trials related to NETs and cancer treatment. **Figure 2** shows cancers in which predominant direction of evidence suggests that NETs degradation could benefit and cancers in which predominant direction of evidence suggests that NETs are involved in the anti-cancer response.

## 2 NETs degradation methods

One of the ways to inhibit the formation of NETs is the administration of Deoxyribonuclease I, Dornase alpha, mainly known as DNase I, which degrades the structure of NETs consisting mainly of DNA (24–26). A change in plasma DNase activity has been linked to the carcinogenesis, as observed in patients with malignant lymphomas, who showed a decrease in its activity, while breast cancer patients showed higher levels of activity compared to healthy subjects (27–29). Even so, physiological amounts of DNase I are not sufficient to completely degrade NETs *in vitro* (30). Meanwhile, as early as 1990, it was discovered that DNase treatment reduced metastasis, but the mechanism of this effect was not understood (15). DNase I also reduced the cell viability of numerous cell lines and prevented tumor cell metastasis to the liver in mice, while the mechanism of these actions was not described (31). DNase I is a Ca^2+/^Mg^2+^-dependent endonuclease distributed in plasma that has the ability to selectively degrade all DNA, including tumor-associated cell-free DNAs (cfDNA) not only DNA associated with NETs (10, 32, 33–35). Therefore, it cannot be ruled out that the examples of anticancer use of DNase presented below are also associated with the removal of DNA that is not necessarily NETs-related (36). The efficiency of NETs degradation depends on the combined activity of two distinct DNases, DNase 1 and DNase 1-like 3 (DNase1L3), which preferentially degrade double-stranded DNA (dsDNA) and chromatin, respectively, and to some extent inhibit the proteolytic activity of NE (37, 38). Elimination of NETs results in no loss of T cells, which restores their anti-tumor activity, reduces early adhesion of tumor cells to NETs, i.e. abolishes the mechanism that causes cancer metastasis (39, 40). Treatment of existing NETs with DNase I also increases the therapeutic efficacy of tumor immunotherapy (12, 41). Unfortunately, long-term use of DNase I is detrimental to the function of the immune defense mechanism, as it increases inflammation through inappropriate release of pro-inflammatory mediators and likely causes increased susceptibility to bacterial infections, a common cause of death among oncology patients (12, 42, 43). Despite the fact that DNase has a beneficial effect on local lesions, it may not be applicable for systemic administration due to its rapid degradation, short half-life, low stability in plasma and limitations in removing protein components from NETs (42, 44). The lack of complete degradation of the protein components of NETs results in less efficacy in abolishing the inflammatory response (45). Also, monomeric G-actin released from neutrophils as a result of NETs formation has the ability to inhibit the enzymatic activity of DNase I, so to achieve its desired effect, high-frequency dosing or other forms of its administration are recommended (46). Raghavan et al. (47) have found that positively charged DNase-loaded particles with a size of 200 nm showed the highest degree of interaction with NETs. To overcome the aforementioned limitations associated with DNase treatment, a growing number of studies have focused on new modes of DNase delivery. For example, Zhu et al. (48) developed a strategy using polyethylene glycol-associated polyamino acids (PAAP) to deliver DNase 1 to prevent liver metastasis in breast and colorectal cancers by degrading NETs. The PAAP/DNase-1 complex degrades chromatin to induce apoptosis, then DNase-1 released into the extracellular space dissociates the NET-DNA complex. The action of this combination is therefore bidirectional, inhibiting both primary tumor growth and potential metastasis (48). Another DNase-delivery structure was developed by Filipczak et al. (49), containing mAb2C5 and DNase I, which together have the ability to self-organize into a micelle-like structure. The 2C5 MDM nanoparticles have the ability to specifically recognize NETs and promote their degradation, containing a monoclonal antibody 2C5 that has strong specificity against nucleohistones, which are found specifically in NETs (49). Yin et al. (50) developed a nanocarrier based on regulating the formation of tumor-associated NETs, which consists of a core of paclitaxel (PTX) and poly-l-lysine (PLL) pro-drug nanoparticles conjugated to an MMP-9 cleavable deoxyribonuclease coating conjugated to Tat I cell penetrating peptide (DNase I), abbreviated as mP-NPs-DNase/PTX, which, when accumulated at a tumor tissue site, can release DNase I in response to MMP-9 to degrade NETs and absorb and dissociate tumor cells. This model enabling DNase I administration *in vitro/vivo* studies increased the inhibition of malignant tumor growth and distant metastasis (50).

NETs formation can also be supressed by inhibiting its major components, which include NE (51, 52). An NE inhibitor, for example, is Sivelestat, whose mechanism of action is to prevent NE nuclear translocation and inhibit chromatin decondensation (24, 53). NE inhibitors also include the leukocyte secretory protease inhibitor (SLPI) and SerpinB1, which limit the production of NETs *in vitro/vivo* (54). Inhibition of peptidylarginine deiminase type 4 (PAD4), the enzyme responsible for the histone modifications required for neutrophil DNA decondensation prior to NETs formation, also has therapeutic indications (15, 55). PAD4 deficiency has been linked to decreased growth of tumors, such as Lewis lung carcinoma (LLC) or pancreatic tumors, and cancer metastasis (56–58). Deletion of PAD4 in neutrophils or pharmacological inhibition of PAD4 with JBI-589 reduced primary tumor growth and lung metastasis, and significantly increased the effect of immune checkpoint inhibitors in mouse models of tumors (59). PAD4 is produced not only by physiological structures such as neutrophils, monocytes, macrophages, brain, uterus, joints, bone marrow, but also by tumorigenesis (60). A major disadvantage of the PAD4 inhibitors used is their serum half-life, as it is only 15 minutes to 4 hours (15). Small-molecule inhibitors of PAD4 include Cl-amidine and F-amidine, which are irreversible inhibitors that bind calcium, which is involved in the formation of NETs, and act by covalent modification at the enzyme’s active site (5, 61). Chlorotetracycline, minocycline and streptomycin were identified as reversible PAD inhibitors with low efficacy, Cl-amidine and F-amidine were formulated as inhibitors with improved efficacy and sensitivity, GSK199 and GSK484 were developed as highly effective selective PAD4 inhibitors (62–64). The aforementioned inhibitors have been used in diseases with comorbid inflammation, where they caused a reduction in inflammation, including autoimmune diseases (62, 65). PAD4 inhibition worked synergistically with the combined checkpoint inhibitors anti-(programmed cell death protein 1, PD-1) and anti-(cytotoxic T-lymphocyte associated protein 4, CTLA-4) (13). Zhu et al. (66) examined modifications of the PAD4 inhibitor with phenylboronic acid (PBA), which has the ability to combine with sialic acid on the tumor surface. The combination showed dual targeting of tumor cells, both from the primary tumor and from metastatic tumors (66). Another route of delivery for the PAD4 inhibitor is its nanocarrier, ZD-E-1. It is formed by self-assembly of a pH-responsive molecular PAD4 inhibitor: ZD-E-1M (67). Most studies show slower tumor growth and/or metastasis after PAD4 inhibition, while there are also studies reporting minimal or no effect, depending on the type of cancer (68).

Zhao et al. (69) developed neutrophil hitchhiking nanoparticles (SPPS) that block NETs formation to enhance Bacteria-mediated tumor therapy (BMTT). In a study in mice, after 24 hours of bacterial therapy, there was an increase in the number of neutrophils in the blood and an increase in SPPS reaching the tumor tissue by stowaway neutrophils (69). The amount of NETs in the tumors decreased by reprogramming the formation of NETs, thereby increasing the viability of the bacteria (69). The researchers also found that the gene drug (siBcl-2) loaded in SPPS can be re-enclosed in apoptotic bodies by reprogramming neutrophils from NETs into apoptosis and allows drug delivery back to tumor cells, further enhancing anti-tumor efficacy with a synergistic effect, resulting in increased tumor inhibition rates and increased survival rates (69).

Anthracyclines, or anticancer antibiotics (e.g., epirubicin, daunorubicin, doxorubicin and idarubicin), acting through DNA intercalation, oxidative stress and topoisomerase II poisoning, inhibit both nicotinamide adenine dinucleotide phosphate (NADPH)-oxidase-dependent and NADPH-oxidase-independent NETs formation *ex vivo* (70, 71). Bystrzycka et al. (72) demonstrated that two antibiotics, azithromycin and chloramphenicol, reduce the release of NETs by modulating the ability of neutrophils to release NETs. Also, NADPH oxidase inhibitors significantly reduce tumor cell invasion, suggesting that it may be mediated by NETs (73). Basyreva et al. (74) found that the anticancer drug, 5-fluorouracil (5-FU) caused a significant and rapid increase in the total number of NETs in the blood, while its shielded nanoscaled polymeric form, amphiphilic poly-N-vinylpyrrolidone (Amph-PVP) nanoparticles, blocked the appearance of NETs in the blood (74). Other drugs that also have the ability to block NETs include diethylcarbamazine, lapatinib, rapamycin, bosutinib, ibrutinib, gentamicin, cyclosporine A, 5-aminosalicylic acid (5-ASA), N-acetyl-l-cysteine (NAC), heparin, Alveofact, Curosurf, methotrexate, hydroxychloroquine, and probiotics (61, 65, 75–79). Metformin, a protein kinase C (PKC) inhibitor used to treat diabetes, also has the ability to reduce the formation of NETs (39). The treatment reduces the components of NETs: elastase, proteinase-3, histones and double-stranded DNA. *In vitro*, metformin prevented DNA release, membrane translocation of PKC-βII and activation of NADPH oxidase in neutrophils, resulting in reduced NETs formation (80). Another drug used to treat diabetes, Exenatide, reduced the formation of NETs both peripheral and originating from lung and colon tumors. It also enhanced the anti-tumor efficacy of PD-1 and CD8+ T-cell blockade by reducing NETs, which induced long-term tumor-protective immunity (81). Another drug for diabetic patients that regulates glycemic fluctuations, Liraglutide, a glucagon-like peptide-1 (GLP-1) induced a reduction in circulating NETs markers MPO, NE and dsDNA by inhibiting reactive oxygen species (ROS) in lung and liver cancer in mice. The drug also enhances the anti-tumor efficacy of PD-1 inhibition, improves IFN-γ release by CD8+ T cells, while this effect could not be observed in the absence of NETs (82).

**Figure 3:**
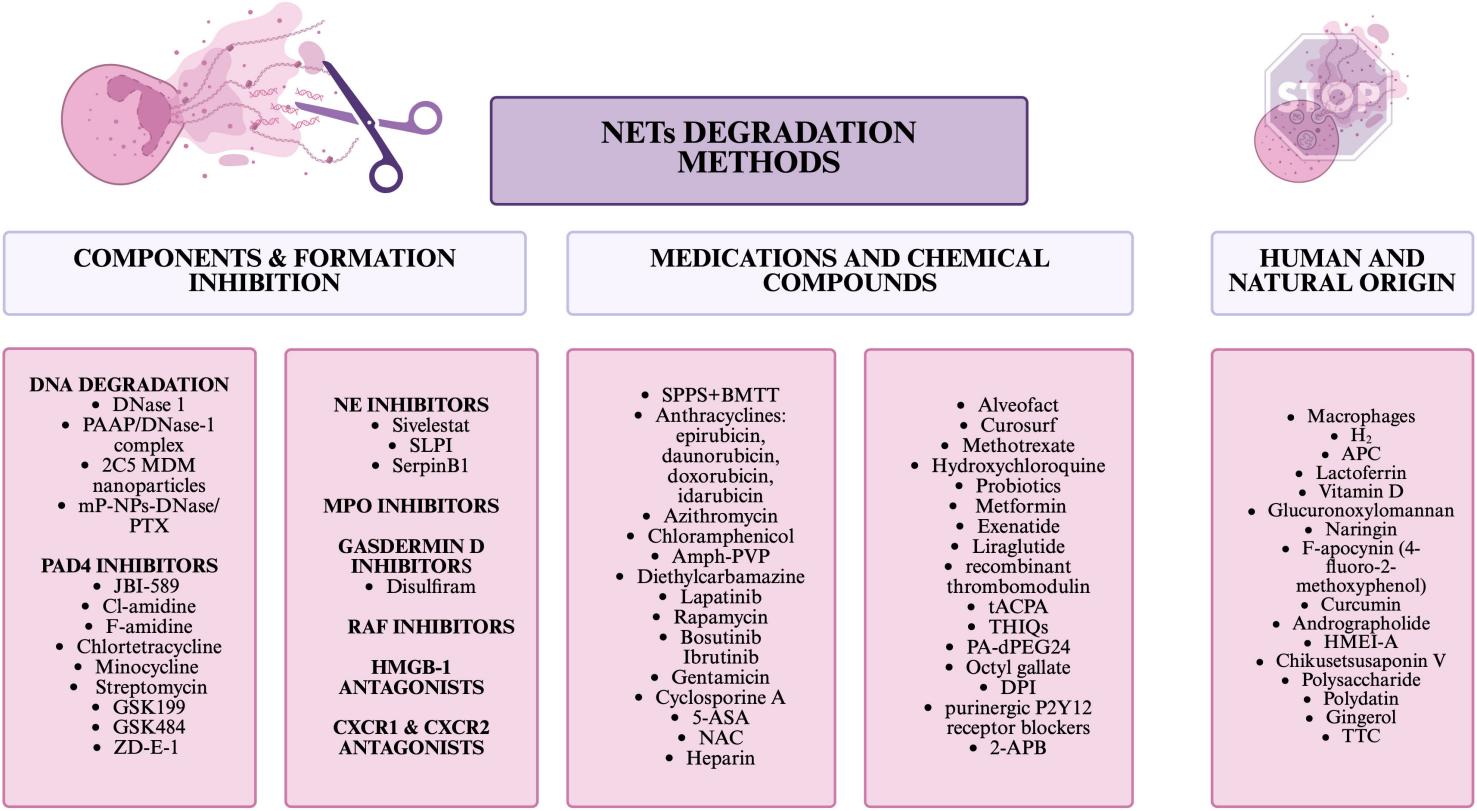
NETs degradation methods.

Interestingly, macrophages also have the ability to degrade NETs *in vivo*, while this has only been studied at this point on human abdominal aortic aneurysm (83). Another way to inhibit NETs is H2 inhalation, which inhibited the formation and release of NETs components in mice and mini-pigs with sepsis (84). Potentially, inhibitors of MPO, a component of NETs, could also be such a compound, but at this point have limited clinical utility due to the side effects they have (22). Disulfiram, which blocks gasdermin D, required for NETs release, also has the ability to block NETs formation. This drug is only approved for the treatment of alcohol abuse disorders due to its effect on aldehyde dehydrogenase (85). There is also a group of compounds that has been linked to inhibiting NETs, while this has not been studied in the context of cancer. Such compounds include: 2-aminoethyl diphenylborinate/2-aminoethyl diphenylborinate (2-APB), PA-dPEG24, lactoferrin, curcumin, Glucuronoxylomannan, Octyl gallate, Diphenyleneiodonium chloride (DPI), F-apocynin (4-fluoro-2-methoxyphenol), CXCR1 and CXCR2 antagonist, High Mobility Group Box-1 (HMGB1) antagonists, purinergic P2Y12 receptor blockers, therapeutic anti-citrullinated protein antibody (tACPA), naringin, vitamin D, tetrahydroisoquinolines (THIQs), Activated protein C (APC), recombinant thrombomodulin, RAF inhibitors (61, 76, 78, 86–94). Also, other substances of natural origin can affect the formation of NETs, among such unstudied for anticancer effects are: Andrographolide derived from *Andrographis paniculata*, HMEI-A derived from *Hirudinaria manillensis*, Chikusetsusaponin V derived from *Panax japonicus*, Polysaccharide derived from *Kochia scoparia*, Polydatin derived from *Polygonum cuspidatum*, Gingerol derived from *Ginger*, and TTC derived from *Celastrus orbiculatus* (95). In **Figure 3**, NETs degradation methods are collected and divided into 3 categories: inhibiting NETs components or their formation, drugs and chemicals, and natural/human substances.

## 3 Predicting efficacy or resistance to anti-cancer therapy associated with NETs. Mechanisms of anti-cancer therapy associated with NETs removal. Anti-cancer effects associated with NETs.

Cell resistance to chemotherapy i.e. chemo-resistance, radiation resistance and resistance to immunotherapy is associated with TME, where neutrophils and their functions play an active role (88, 96). Massive neutrophil infiltration is often associated with poorer response to antitumor therapy, as has been demonstrated in several different types of cancer (96–98). In chemotherapy-resistant patients who are unlikely to benefit clinically, treacnt may cause side effects related to drug toxicity or delay the use of other effective treatments, hence it is important to understand the mechanisms responsible for chemoresistance, and one of these potential mechanisms may include NETs (99–102).

Cancer cells that die, often as a result of therapy, release adenosine triphosphate (ATP), which induces NLR family pyrin domain containing 3 (NLRP3) activation in surviving cancer cells, which then leads to the release of interleukin 1-beta (IL-1β), which in turn can stimulate the formation of NETs (103). NETs have the ability to transform TME by reducing the number of anti-tumor effector cells, which can impair the efficacy of immunotherapy (104). For example, NETs affect tumor-infiltrating T cells by determining the response to immune checkpoint inhibitors (62). Teijeira et al. (13) described that inhibition of NETs sensitizes tumors to dual anti-PD-1+ anti-CTLA-4 checkpoint blockade. Volkov et al. (105) suggest that by reducing the number of NETs in TME, the efficacy of CAR-T (T-cells modified by the chimeric antigen receptor) therapy can be increased and even extended to solid tumors. Cheng et al. (106) developed the Tandem-locked NETosis Reporter 1 (TNR1), which activates fluorescence signals only in the presence of both NE and cathepsin G (CTSG) to specifically image NETosis and distinguish it from neutrophil activation. Near-infrared signals from activated TNR1 correlated negatively with the effect of tumor suppression after immunotherapy, thus providing a prognosis for cancer immunotherapy (106).

### 3.1 Head and neck cancer

#### 3.1.1 Head and neck squamous cell carcinoma

##### 3.1.1.1 NETs-related predicting efficacy of anti-cancer therapy

Li et al. (107) developed a NETs-related gene signature strongly associated with clinicopathologic and immunologic features of patients with head and neck squamous cell carcinoma (HNSCC). HNSCC patients with low NETs signatures tended to express higher levels of immune checkpoints, including CD274 and CTLA4, and responded better to targeted therapies using afatinib, erlotinib, ibrutinib and lapatinib. In contrast, patients with high NETs signatures were more likely to fail to respond to immunotherapy (107). Anti-PD-1, anti-CTLA4, or combination immunotherapy was more beneficial in patients with low-risk HNSCC stratified by a risk model consisting of six NETs-related genes. Response to anti-cancer drugs was also closely correlated with the expression of NETs-related genes (108).

##### 3.1.1.2 NETs anti-cancer effects presented on human

The largest subgroup of CD16^high^ CD62L^dim^ neutrophils found in HNSCC patients had an increased ability to migrate and to form NETs, but was equally associated with anti-tumor effects and increased survival in HNSCC patients (109).

### 3.2 Central nervous system tumors

#### 3.2.1 Glioblastoma multiforme

##### 3.2.1.1 NETs-related predicting efficacy of anti-cancer therapy

Sun and Liu (110) developed a prognostic model based on NETs that enables the selection of precise targeted therapy for glioblastoma multiforme. With the model, patients were divided into two groups, where patients in the high-risk group were more sensitive to bicalutamide, dasatinib and gefitinib, while patients in the low-risk group were associated with a poor response to immunotherapy (110).

#### 3.2.2 Glioma

##### 3.2.2.1 NETs degradation anti-cancer effects presented on cell lines and human tissues

NETs produced by tumor-infiltrating neutrophils (TINs) mediate the communication between glioma progression and TME by regulating the HMGB1/RAGE/IL-8 axis (111). HMGB1 derived from NETs binds to RAGE and activates the nuclear factor κB (NF-κB) signaling pathway, which is also stimulated by NETs and promotes interleukin 8 (IL-8) secretion in glioma. IL-8 then recruits neutrophils, which in turn mediated NET formation through the PI3K/AKT/ROS axis. Overall, overproduction of NETs promoted the proliferation, migration and invasion of glioma cells, with a greater number of NETs detected in high-differentiation gliomas compared to low-differentiation gliomas. NETs promoted the rapid proliferation of glioma cells and their ability to invade, while this effect was abolished by DNase I. Thus, targeting NETs formation or IL-8 secretion may be an effective approach to inhibit glioma progression (111).

### 3.3 Respiratory tract cancers

#### 3.3.1 Non-small cell lung cancer

##### 3.3.1.1 NETs-related predicting efficacy of anti-cancer therapy

A study by Guo et al. (112) indicates that serum NETs levels are an effective predictor of PD-1 inhibitor response used in the treatment of advanced non-small cell lung cancer (NSCLC) and reflect the neutrophil-to-lymphocyte ratio (NLR) in the tissue and the likelihood of immune-related adverse events (IrAEs). Lower serum NETs concentrations have been associated with better immunotherapeutic effects. The combination of serum NETs, CD8+ T cells and tumor proportion score (TPS) predicted the efficacy of PD-1 inhibitor treatment (112).

#### 3.3.2 Lung cancer

##### 3.3.2.1 NETs degradation anti-cancer effects presented on cell lines

Najmeh et al. (40) conducted studies on lung cancer cell lines, which showed that administration of DNase 1 caused a decrease in cancer cell adhesion and that integrins can mediate cancer cell interactions with NETs. DNase I or an NE inhibitor also abolished the formation of hepatic micrometastases formed by the transfer of lung cancer cells by NETs (16, 113).

##### 3.3.2.2 NETs degradation anti-cancer effects presented on mice

Sun et al. (11) developed a hybrid nanoparticle composed of DNase I and gold (DNase I@Au) administered inhaled to increase the efficacy of radiotherapy, used to treat lung cancer, and to increase the elimination of NETs that promote metastasis. The nanoparticle reduces tumor size, gradually releases DNase thereby degrading NETs, preventing free malignant cells from interacting with tumor sites or blood vessels. The molecule tested supressed the formation of breast cancer metastases to the lungs (11).

### 3.4 Gastrointestinal cancers

#### 3.4.1 Esophageal cancer

##### 3.4.1.1 NETs degradation anti-cancer effects presented on cell lines

The MPO inhibitor azide reduces increased levels of neutrophil ROS leading to mucosal damage in Barrett’s esophagus, considered a precancerous lesion of esophageal adenocarcinoma. In contrast, Sivelestat sodium, a type of NE inhibitor, can attenuate postoperative complications in esophageal cancer patients: it significantly reduces postoperative hypoxia, partially reduces systemic inflammation and maintains postoperative circulatory status (114).

#### 3.4.2 Gastric cancer

##### 3.4.2.1 NETs-related predicting efficacy of anti-cancer therapy

Li et al. (115) discovered that sensitivity to chemotherapeutic treatment of gastric cancer was linked to the expression of NETs-related genes, from which a potential prognostic risk score “NETs-Score” was created. The study groups were divided into those at “low risk” and those at “high risk.” The researchers used 3 immune checkpoints to assess the potential efficacy of the therapy: CTLA-4, PD-1 and programmed death ligand 1. As a result, they found that there were significantly more of them in the low-risk group, meaning this group was more likely to activate immune defenses and respond to immunotherapy (115). Low risk was associated with lower inhibitory concentrations (IC_50_) of chemotherapeutics such as afatinib, dactinomycin, daporinad, docetaxel, ibrutinib, lapatinib, sepantronium bromide and 5-FU. The NETs-Score acted as a potential predictor of chemosensitivity (115). Yang et al. (116) created a prognostic model for gastric cancer using long non-coding RNA (lncRNA) associated with NETs, which demonstrated prognostic capabilities, serving as an adjunct to traditional cancer staging and enabling the selection of an appropriate treatment option. The researchers also analyzed checkpoint genes, which were found to be strongly expressed in the high-risk group, while only two genes, TNFRSF14 and LGALS9, were strongly expressed in the low-risk group (116). They also conducted an analysis of the relationship between risk score and drug resistance, which showed that the IC_50_ value of dasatinib was higher in the low-risk group, while the sensitivity of other targeted drugs, namely AZD5363, dabrafenib, GSK269962A, ipatasertib, lapatinib, MK-2206, oxaliplatin, palbociclib, PF-4708671, ribociclib, ulixertinib, VE-822 in the low-risk group was higher than in the high-risk group (116). Zhang et al. (117) demonstrated that NETs in gastric cancer activate cyclooxygenase-2 (COX-2) through Toll-like receptor 2 (TLR2) which increases the metastatic capacity of cancer cells. The correlation of COX-2 with NETs was confirmed by the use of DNase I, and mice given it showed lower COX-2 levels and delayed metastasis (117). Moreover, COX-2 was correlated with anti-CTLA4 response and a group of gastric cancer patients with high COX-2 levels showed lower sensitivity to afatinib, erlotinib, gefitinib, ibrutinib, osimertinib, Wnt-C59, AZD1332, AZD3795, CDK9, P22077 and XAV939 (117). An interesting relationship between the efficacy of advanced first-line treatment of gastric cancer depending on the level of NETs was presented by Zhang et al. (118). In patients with a partial response to treatment, patients with stable disease and controls, the levels of NETs before treatment were higher than after treatment in both plasma and serum. In contrast, in patients with progressive disease, NET levels before treatment were lower than after treatment in both plasma and serum (118).

##### 3.4.2.2 NETs degradation anti-cancer effects presented on cell lines and mice

Tao et al. (119) discovered that Danshen, a dried root of *Salvia miltiorrhiza* known for its anticancer properties, among other things, reduces lung metastasis of gastric cancer cells. The mechanism of this action takes into account the prevention of the movement of neutrophils to metastatic sites with reduced NE levels. Danshen-derived compounds salvianolic acid B (Sal B) and 15,16-dihydrotanshinone I (DHT I) have shown inhibitory effects on the formation of NETs by acting on MPO and NOX (119). In *in vitro* studies, after treatment with phorbol myristate acetate (PMA), which promotes NETs formation, or DNase 1/GSK-484, which inhibits NETs formation, the ability of gastric tumors to migrate was found to be altered; however, no significant changes were observed in cell proliferation or cell cycle progression (120).

##### 3.4.2.3 NETs anti-cancer effects presented on mice

Ju et al. (121) developed a neoadjuvant chemotherapy based on Abraxane/human neutrophils cytopharmaceuticals together with radiotherapy to treat gastric cancer. In this regimen, neutrophils are used to carry Abraxane, a commercial albumin-bound PTX nanoparticle that maintains the intrinsic function of neutrophils. Radiotherapy increases the release of inflammatory factors that increase the influx of neutrophils into the tumor area, NETs are formed, resulting in the shedding of Abraxane and improved tumor suppression (121).

#### 3.4.3 Hepatocellular carcinoma

##### 3.4.3.1 NETs-related predicting efficacy of anti-cancer therapy

Hepatocellular carcinoma (HCC) cell resistance to drugs and tumor sensitivity to chemotherapeutics showed a significant correlation with the expression of prognostic NETs-related genes (NETs) (122). Yuan et al. (123) constructed a six-gene NETs-related signature that could predict survival outcomes in patients with HCC. The TME of HCC differed between high-risk and low-risk groups, which influenced tumor resistance to therapy. Researchers used the Immunophenoscore (IPS) scale to assess susceptibility to immunotherapy in high- and low-risk subgroups. In the high-risk group, most immune-related genes were poorly expressed, while the low-risk subgroup showed a higher IPS in the CTLA4-PD1+, CTLA4+ PD1- and CTLA4+ PD1+ groups (123). Higher IPS indicated a more favorable immunotherapeutic response, with those in the low-risk group showing an increased response to immunotherapy. When analyzing the response to another therapeutic strategy, chemotherapy, the IC_50_ values of 9 drugs: A-443654, AKT VIII inhibitor, PD-173074, BMS-509744, CCT007093, CGP-60474, GSK690693, JNK-9L and KIN001-102, showed a marked reduction in the high-risk group compared to their low-risk counterparts, indicating increased sensitivity to treatment (123). In other studies as well, the group with low expression of NETs-related genes showed higher expression levels of immune checkpoint genes, so they tended to respond better to immunotherapy compared to the group with high expression of NETs-related genes (124).

#### 3.4.4 Liver cancer

##### 3.4.4.1 NETs degradation anti-cancer effects presented on cell lines

The ability of neutrophils to stimulate invasion tested on the human liver cancer cell line HuH7 was inhibited by DNase I, while it showed no effect on tumor cell invasion stimulated with fetal bovine serum (FBS). Pre-incubation of neutrophils with the PAD4 inhibitor GSK484 before co-culture reduced the ability of neutrophils to form NETs, which in turn blocked the promotion of HCC cell invasion (125). The neutrophils tested could significantly increase the trans-endothelial migration of HepG2 cells, while this effect was abolished by DNase I (126).

##### 3.4.4.2 NETs degradation anti-cancer effects presented on mice

Mou et al. (127) investigated the suppressive effects of icaritin (ICT), used to treat HCC in mice. ICT inhibited the growth of subcutaneous tumors, increased infiltration of CTLs, macrophages and M1-type macrophages, and promoted the secretion of anti-tumor effector molecules such as IFN-γ and Granzyme B (127). Inside the tumor, researchers found ICT-induced suppression of neutrophil infiltration. Reduction of NETs by DNase I or PAD4 inhibitor, could inhibit HCC tumor metastasis in mice *in vivo* (126). Also, in a study by Yang et al. (45) in mice, combining DNase 1 with the anti-inflammatory drugs aspirin/hydroxychloroquine (HCQ) effectively reduced hepatocellular carcinoma metastasis. NETs trigger an inflammatory response in trapped HCC cells. Treatment with prostaglandin E2 (PGE2), a direct product of COX2, abolished the effects of NETs on HCC cells. HCQ, a drug with the ability to block the TLR pathway, can effectively abolish the up-regulation of COX2 and subsequently block the metastatic behavior of HCC cells induced by NETs (45). In a study by Zhan et al. (128) DNase 1 also inhibited the growth and lung metastasis of hepatocellular carcinoma induced by NETs. Acting on oxidized mitochondrial DNA (mtDNA) with metformin prevents HCC metastasis enabled by NETs. HCC cells are able to stimulate the formation of NETs rich in oxidized mtDNA, which have strong pro-inflammatory and metastasis-promoting properties. Metformin treatment reduced the formation of NETs, decreased the up-regulation of several inflammatory mediators that promote metastasis triggered by HCC-NETs, i.e. reduced the inflammatory response accompanying the tumor (129).

##### 3.4.4.3 NETs degradation anti-cancer effects presented on cell lines and mice

NETs-CM, a conditioned medium containing NETs, markedly increased the invasive potential of HCC cells. Co-culture of NETs-CM with a cathepsin G inhibitor equally blocked the ability to induce invasion. Digestion of NETs-DNA by DNase I prevented invasion, although CTSG was not removed by DNase I digestion. The NE inhibitor, Sivelestat, showed no significant effect on neutrophil-stimulated invasion. In contrast, in *in vivo* studies in mice, NETs-CitH3 complexes began to be detectable at the pre-metastatic stage in a model lung compared to controls, which could be abolished by DNase I treatment. The cathepsin G inhibitor showed little effect on NETs formation *in vivo*, while it significantly reduced NETs-CitH3 release *in vitro*. NETs-derived CTSG promoted HCC cell invasion by reducing E-cadherin expression *in vitro* (125). Yoshimoto et al. (130) demonstrated that NETs promoted the motility and migratory capacity of intrahepatic cholangiocarcinoma (iCCA) cells *in vitro*. The increased motility of cancer cells after co-culture with NETs was abolished by DNase and the PAD4 inhibitor, Cl-amidine. The co-culture was also characterized by decreased expression of E-cadherin and increased expression of vimentin. P-selectin-mediated platelet binding to tumor cells promoted the induction of NETs, an effect that was abrogated by the use of antiplatelet drugs. Injection of iCCA cells into the spleen of mice induced liver micrometastases coexisting with NETs. Reduction of metastasis was achieved after treatment with dual antiplatelet therapy (DAPT) consisting of aspirin and ticagrelor (130).

##### 3.4.4.4 NETs degradation anti-cancer effects presented on mice and rabbits

Cheng et al. (32) formulated a dual pH-responsive hydrogel with a tumor acidity neutralizer in the form of mesoporous bioactive glass nanoparticles and DNase I, which they used in combination with infusion of NK cells, which have the ability to selectively recognize and kill cancer cells. The combination of NK cell infusion and a hydrogel-based delivery system can effectively prevent HCC recurrence after resection. NK cell infusion is negatively affected by acidic TME and NETs, so combining with a biocompatible hydrogel that neutralizes tumor acidity and leads to NETs lysis would significantly improve the efficacy of the therapy. The gel also had the ability to reduce tumor infiltration by M2-type macrophages, regulatory T cells and MDSCs and to activate endogenous anti-tumor immunity associated with CD8+ T cells (32).

#### 3.4.5 Pancreatic carcinoma, pancreatic cancer, pancreatic adenocarcinoma & pancreatic ductal adenocarcinoma

##### 3.4.5.1 NETs-related predicting efficacy of anti-cancer therapy

Zhang et al. (3) created a prognostic model based on NETs and (epithelial-mesenchymal transition) EMT signatures in patients with pancreatic adenocarcinoma (PAAD), the use of which indicates potentially effective immunotherapeutic strategies and can predict the prognosis of patients with PAAD. This prognosis was strongly correlated with immune invasion, immune cycle, immune checkpoint and sensitivity to treatment. NETs are promising potential targets for neoadjuvant immuno- and chemotherapy against cancer metastasis in patients with PAAD. In addition, a combined suppressor of NETs and EMT may be a highly effective intervention for patients with PAAD (3). Interleukin 17 (IL-17) sustains pancreatic ductal adenocarcinoma (PDAC) immunosuppression by reducing CD8+ T-cell recruitment and activation, and recruits neutrophils and stimulates NETs formation in pancreatic tumors *via* factors released from tumor cells. IL-17 blockade increased sensitivity to PD-1 and CTLA4, while blockade of neutrophils or PAD4-dependent NETs formation synergized with PD-1 blockade to dramatically reduce tumor growth (2). PDAC patients with lower neutrophil infiltration, where 45.4% have the ability to form extracellular traps or negative staining for neutrophil extracellular traps, are more likely to benefit from adjuvant chemotherapy (131).

##### 3.4.5.2 NETs degradation anti-cancer effects presented on cell lines

A study by Deng et al. (73) showed that inhibition of PAD4 and NE inhibited NETs formation and tumor cell invasion in neutrophils co-cultured with a primary human PDAC cell line with strong expression of discoid domain receptor 1 (DDR1) and a cell line without DDR1 expression. NADPH oxidase inhibition had no effect on NETs or tumor cell invasion, and DNase I treatment showed only a partial effect compared to the control group. NETs formation, phosphorylation of NF-κB, PKC and SYK, CXCL5 production, and cancer cell invasion were significantly reduced in cells treated with 7rh benzamide, a specific DDR1 inhibitor. The researchers suggest that administration of this inhibitor at therapeutic doses, may increase the sensitivity of cancer cells to conventional chemotherapy and inhibit liver metastasis by blocking the formation of NETs (73).

##### 3.4.5.3 NETs degradation anti-cancer effects presented on mice

Takesue et al. (132) discovered that DNase I, by inhibiting NETs, suppressed PDAC metastasis to the liver. DNase I also inhibited micrometastasis and reduced the number of Cancer-associated fibroblasts (CAFs), a major component of TME in PDAC. In PDAC, pancreatic cancer cells induce the formation of NETs, which increase the migration of hepatic stellate cells, a source of CAFs potentially involved in metastasis formation (132). In another studies, DNase treatment of mice reversed the ability of pancreatic stellate cells (PSCs) to promote tumor growth, as demonstrated by the reduced tumor weight of treated mice. DNase’s mechanism of action involved blocking endogenous DNA, derived from NETs, which had the ability to activate PSCs (57).

##### 3.4.5.4 NETs degradation anti-cancer effects presented on mice and human tissues

Canè et al. (133) demonstrated that NETs present in PDAC patients form a microdomain in which cathepsin S (CTSS) cleaves human arginase 1 (hARG1) into different molecular forms endowed with enhanced enzymatic activity at physiological pH. Arginase 1 (ARG1) has the ability to degrade arginine, which inhibits the anti-tumor response (133). NETs-associated hARG1 inhibits T cells, whose proliferation can be restored by adding a monoclonal antibody (mAb) specific for hARG1 or by preventing CTSS-dependent cleavage (133). Researchers have found that ARG1 blockade, in combination with immune checkpoint inhibitors, can restore CD8+ T cell function in PDAC tumors *ex vivo*, and that anti-hARG1 monoclonal antibodies increase the number of tumor-specific CD8+ T cells in the tumor and enhance the efficacy of immune checkpoint therapy in humanized mice (133). Wang et al. (134) discovered that metformin could effectively inhibit the progression of pancreatic cancer promoted by obesity, where adipocytes promoted NETs formation, a phenomenon that did not occur in lean mice. NETs promote pancreatic carcinogenesis through activation of TLR4-dependent pathways, expression of inflammatory factors and initiation of EMT. In a study, metformin and DNase I significantly reversed the pro-cancer effects of obesity and NETs *in vitro/vivo*. DNase I inhibited the progression and EMT of pancreatic intraepithelial ductal neoplasia in mice, while metformin suppressed the inflammatory response induced by NETs in these cells manifested as increased IL-1β expression (134). Kajioka et al. (44) described that thrombomodulin degraded HMGB1, which inhibited NET induction, thereby preventing pancreatic cancer metastasis to the liver, and blocked EMT and attenuated the malignant potential of pancreatic cancer cells. Researchers examined that mice with NETs that were given DNase had significantly reduced liver metastasis. The finding has implications for surgical procedures performed to treat pancreatic cancer, which promote liver metastasis and often cause systemic inflammation, leading to NETs (44).

##### 3.4.5.5 NETs degradation anti-cancer effects presented on mice and human

In a study by Boone et al. (135) inhibition of autophagy by chloroquine treatment reversed the propensity to form NETs *in vitro*. Both mouse models and patients treated by inhibiting autophagy had reduced NETs formation both by circulating neutrophils and in TME PDAC. Moreover, the greater the response to treatment, the more effective inhibition of NETs occurred in TME (135).

##### 3.4.5.6 NETs anti-cancer effects presented on mice

Chan et al. (136) presented beneficial anti-tumor effects of NETs in patients with pancreatic adenocarcinoma. Melatonin supplementation induced neutrophils and increased the occurrence of NETs, resulting in apoptosis of tumor cells via cell-to-cell contact. The number of NETs increased during melatonin treatment, resulting in slower tumor growth (136).

#### 3.4.6 Colon carcinoma

##### 3.4.6.1 NETs-related predicting efficacy of anti-cancer therapy

The results obtained by Feng et al. (104) indicate that the prognostic signature of six NETs-related genes, CRISPLD2, CPPED1, VNN3, ENTPD4 and MPO, can estimate the prognosis and response to chemo-/immunotherapy in patients with colon carcinoma (COAD). Researchers used the Tumor Immune Dysfunction and Exclusion (TIDE) technique to assess response to immunotherapy. The technique is able to predict immunotherapeutic response based on two main mechanisms of tumor immune escape: infiltration and T-cell dysfunction. The higher the TIDE score, the stronger the potential for immune evasion, i.e., the more likely patients are to benefit from immune checkpoint inhibitor therapy. Compared to a high-risk NET population with high TIDE scores, a better prognosis can be obtained for a low-risk NET population with low TIDE scores (104).

##### 3.4.6.2 NETs degradation anti-cancer effects presented on mice

Systemic treatment with DNase I and a mixture of proteases in rats with colorectal cancer showed antitumor effects, reduced the amount of DNA and proteins in serum. Researchers did not observe anti-cancer effects in immunodeficient mice treated with enzymes administered separately (137).

#### 3.4.7 Colorectal cancer

##### 3.4.7.1 NETs-related treatment resistance

In a study on colorectal cancer mice, DNase I degraded NETs induced by tumor cells, suppressing NETs-created resistance to anti-PD-1 blockade by increasing CD8+ T-cell infiltration and cytotoxicity. In addition, it reduced the number of tumor-associated neutrophils (138). Wang et al. (139) discovered that the PAD4 inhibitor, GSK484, promotes colorectal cancer (CRC) radiosensitivity and inhibits the formation of NETs both *in vitro*/vivo. Researchers detected PAD4 overexpression in CRC patients, which was also an indicator of adverse disease prognosis. GSK484 treatment promoted tumor cell radiosensitivity, induced cell death by promoting DNA double-strand breaks, inhibited the effects of PAD4 overexpression in irradiated cells, and inhibited the formation of NETs *in vivo* (139). Chen et al. (12) designed a plasmonic core black-body gold (AuPB) nanoplatform with a broad spectrum of photoactivity and a mesoporous polydopamine (mPDA) coating for efficient loading and photo-regulated release of DNase I. The on-demand DNase I released by the mechanism triggered by a second near-infrared light irradiation (NIR-II) breaks down the barrier formed by NETs, thereby increasing the contact of immune cytotoxic cells with tumor cells in living mice and sensitizing CRC to immune checkpoint therapy. Moreover, the use of this mechanism in the liver, the most common site of CRC metastasis, abolished NETs-mediated metastatic spread. Also, the anti-tumor therapeutic effect of the PD-1 monoclonal antibody was enhanced by DNase I delivery (12). In patients with locally advanced rectal cancer treated with neoadjuvant therapy, a high density of NETs in biopsy specimens was significantly associated with a decreased likelihood of a complete/proximal tumor response to therapy (140).

##### 3.4.7.2 NETs degradation anti-cancer effects presented on cell lines

In a study by Wang et al. (141) mice with colorectal cancer treated with DNase I after injection of lipopolysaccharide (LPS) to stimulate NETs formation showed significantly less metastasis compared to mice treated with LPS alone, which was also associated with a decrease in the expression of TLR9, p-p38, p-p65, p-JNK and p-Stat, and the same effect could be observed after using the PAD4 inhibitor YW4-03 (141).

##### 3.4.7.3 NETs degradation anti-cancer effects presented on mice

In a study conducted by Zhang et al. (138) combination therapy for CRC with DNase I and PD-1 antibody showed higher efficacy, prevented tumor growth to a greater extent compared to treatment with a single agent *in vitro/vivo*. Due to the limitations of DNase administration, Xia et al. (46) developed a new startegy for its delivery, a gene therapy vector based on an adeno-associated virus (AAV) that specifically expresses DNase I in the liver, which would reduce the development of liver metastasis by modulating the innate and adaptive immunity of colorectal tumors. In a study conducted on mice with CRC, the developed therapeutic startegy inhibited the development of liver metastases, reduced neutrophil infiltration into the tumor and the formation of NETs, while the percentage of CD8+ T cells increased (46). Pan et al. (142) showed that Huang Qin Decoction inhibits intestinal tumor initiation and proliferation by attenuating inflammation, i.e. by reducing intestinal neutrophil infiltration, enhancing CD8+ T-cell immune surveillance, and by controlling NETs formation through effects on PAD4. Reduced levels of interleukin 1 (IL-1), tumor necrosis factor α (TNF-α) and MMP-9, alleviation of decreased intestinal permeability caused by intestinal damage, and elevated white blood cell and granulocyte counts after decoction were noted in the mice studied (142). Rayes et al. (143) found that blocking carcinoembryonic Ag cell adhesion molecule 1 (CEACAM1) associated with NETs leads to a significant reduction in adhesion, migration and metastasis of colorectal cancer cells. NETs-associated CEACAM1 promotes colorectal cancer cell adhesion and migration *in vitro/vivo*, and increases the possibility of metastasis formation *in vivo* (143).

##### 3.4.7.4 NETs degradation anti-cancer effects presented on cell lines and mice

In a study by Yazdani et al. (144) both in mice lacking PAD4, and therefore unable to form NETs, and in non-PAD4-deficient mice, DNase reduced tumor-associated inflammation and reduced metastatic tumor growth in the liver. Inhibition of NETs formation by DNase and NE inhibitor (NEi) *in vivo* or blocking the NE-TLR4-PGC-1α axis *in vitro* can inhibit mitochondrial biogenesis and slow tumor growth (144). Inhibition of CRC metastasis formation in the liver by NE inhibition with Sivelestat was also confirmed by a mouse study by Okamoto et al. (145). Blocking NE is an effective option due to the release of NE during the formation of NETs, which accelerates CRC cell migration through activation of ERK *in vitro* which is important in cell proliferation, differentiation and migration, and enables infiltration of tumor cells from veins into liver tissues, which is the initial step in liver metastasis (145). Tohme et al. (146) found that in patients undergoing liver resection for metastatic CRC, increased postoperative NETs formation was associated with a more than 4-fold reduction in disease-free survival. NETs formation increases in response to the stress of surgery, which correlates with accelerated development and progression of metastatic disease. These effects were abolished in mice by local DNase treatment or PAD4 inhibition (146). Inhibition of PAD4-enabled citrullination by the PAD4 inhibitor, BB-Cl-amidine, significantly reduces the burden of CRC metastasis to the liver, where higher levels of PAD4 were observed compared to healthy liver and primary tumor (147). IFNγ treatment on cell lines from patients with Microsatellite Stable Colorectal Cancer induced more NETs formation and cell apoptosis. The results were confirmed in mice with this tumor, where IFNγ reduced tumor size and increased tumor killing activity induced by PD-1 antibody, accompanied by increased NETs formation and cell apoptosis (148).

##### 3.4.7.5 NETs degradation anti-cancer effects presented on human tissues and serum

Due to the increased expression levels of NE and its ability to generate an environment favorable to tumor cells by degrading the insulin receptor substrate-1 (IRS-1) and increasing the interaction of phosphatidylinositol 3-kinase (PI3K) and the potent platelet-derived growth factor mitogen receptor (PDGF) in CRC patients, Ho et al. (149) have proposed a potential therapeutic strategy for this cancer involving blocking the enzymatic activity of NE using Sivelestat to inhibit tumor progression. The results indicate that Sivelestat can inhibit tumor growth (149). Zhang et al. (150) demonstrated that epigallocatechin-3-gallate (EGCG), one of the main active components of tea catechins, inhibits the formation of NETs, consequently suppressing the migration and invasion of colon cancer cells by regulating the signal transducer and activator of transcription 3 (STAT3)/CXCL8 (IL-8) signaling pathway. Compared to healthy subjects, STAT3 and CXCL8 mRNA expression was increased in neutrophils from colorectal cancer patients, as was STAT3, p-STAT3 and CXCL8 protein expression (150). Overexpression of STAT3 promoted CXCL8 production and NETs formation in colorectal cancer patients (150). STAT3 deficiency, like DNase I, inhibited NETs formation (150). EGCG treatment inhibited STAT3 and CXCL8 expression and NETs formation in colorectal cancer-derived neutrophils (150).

##### 3.4.7.6 NETs anti-cancer effects presented on mice and human

NETs can limit the growth of CRC cells *in vitro* by inducing apoptosis and/or inhibiting proliferation. Interestingly, the use of DNase I or heparin abolished the inhibitory effect (151). Chemotherapy for CRC produces NETs that release cathepsin G, which enters cancer cells and induces apoptosis. Specifically, the combination of the glutaminase inhibitor CB-839 and 5-FU inhibited the growth of colorectal cancers with PIK3CA mutation in part through NETs in mouse models. Degradation of NETs by DNase I or deletion of neutrophils attenuated the anti-tumor effect of the drug combination tested. The mechanism of this action was the induction of IL-8 expression preferentially in CRC with PIK3CA mutation to attract neutrophils to tumors, increasing ROS levels in neutrophils, inducing NETs. CTSG, a component of NETs, enters CRC cells through the cell surface protein RAGE, where it cleaved 14-3-3ϵ proteins, causing mitochondrial translocation of BAX and inducing apoptosis in CRC cells. Researchers conducted a phase II clinical trial of the combination of CB-839 and capecitabine, an oral pro-drug of 5-FU, in patients with metastatic colorectal cancer with a PIK3CA mutation who were refractory to prior fluoropyrimidine-based chemotherapy, which showed an increased number of NETs in most patients’ tumors, which was associated with longer progression-free survival. These patients also showed reduced tumor growth, but no more than 30% (152).

### 3.5 Urological cancers

#### 3.5.1 Clear cell renal cell carcinoma

##### 3.5.1.1 NETs-related predicting efficacy of anti-cancer therapy

NETs gene signatures were significantly correlated with the sensitivity of clear cell renal cell carcinoma (ccRCC) to targeted therapy with afatinib, axitinib, erlotinib, gefitinib, ibrutinib and saptinib. With the exception of TIM-3, the expression of most selected immune checkpoints, namely PD-1, CTLA4, LAG3, A2BR and B7-H3, was significantly increased in the high-risk group (153). Quan & Huang (154) identified 23 NETs-related genes in ccRCC and three clusters of ccRCC cases with significant differences in disease prognosis, immune infiltration and response to chemotherapy, specifically to axitinib, cisplatin, gemcitabine, sorafenib and sunitinib and targeted therapy. The signature of 6 NETs-related genes, G0S2, DYSF, MMP9, SLC22A4, SELP and KCNJ15, was significantly correlated with drug sensitivity in ccRCC patients (154). NETs levels in tumor tissue can also predict treatment efficacy in patients with metastatic ccRCC who have received systemic therapy. Elevated levels of NETs in tumor tissue have also been associated with poor efficacy in increasing patient survival (155).

#### 3.5.2 Bladder cancer

##### 3.5.2.1 NETs-related predicting efficacy of anti-cancer therapy & nets-related treatment resistance

A high NETs-score, has been associated with poor response to chemotherapy and reduced recurrence-free survival of patients with muscle-invasive bladder cancer (MIBC) (99). In a bladder cancer model Shinde-Jadhav et al. (24) observed increased deposition of NETs in the TME in mice after radiation therapy. Inhibition of NETs, via DNase I or NEi, improved the response to radiation. NETs were observed in MIBC tumors in patients who did not respond to radiation therapy or had chronic disease after treatment. HMGB1-dependent induction of NETs in the context of radiotherapy is mediated by Toll-like receptor 4 (TLR4). In *in vivo* studies, inhibition of both HMGB1 and NETs delayed tumor growth (24).

##### 3.5.2.2 NETs degradation anti-cancer effects presented on human

Patients with bladder cancer were characterized by increased formation of NETs both systemically and in the TME, partly due to impaired DNase I-mediated degradation of NETs. The degradation defect can be therapeutically restored *in vitro* with recombinant human DNase (rhDNaseI), Pulmozyme^®^. Compensation of DNase I downregulation, associated with reduced formation of NETs in TME reduces the likelihood of tumor progression and metastasis (25).

##### 3.5.2.3 NETs anti-cancer effects presented on mice

Bacillus Calmette-Guerin (BCG), a treatment for bladder cancer, induces the formation of NETs, which in turn had cytotoxic effects, induced apoptosis and cell cycle arrest in the G0/G1 phase, and inhibited the migration of tumor cells into the bladder environment (156, 157). Mean tumor weight and volume were lower in mice given NETs. The effect of NETs was almost eliminated by protein inactivation, while increased intratumor CD3+ and CD14+ infiltration was reduced by boiling, but not by DNase pretreatment (156).

#### 3.5.3 Urothelial cancer

##### 3.5.3.1 NETs degradation anti-cancer effects presented on cell lines and mice

In a study by Mou et al. (127) ICT, a metabolite of icariin, a Chinese herbal remedy, reduced the production of NETs by the suicide pathway and prevented neutrophil infiltration into the microenvironment of urothelial carcinoma. The mechanism of action involves ICT binding to protein-arginine deiminase 2 (PADI2) in neutrophils and inhibiting granulocyte-macrophage colony-stimulating factor (GM-CSF), interleukin 6 (IL-6) expression and inhibiting PADI2-dependent histone citrullination. ICT enhances the infiltration of cytotoxic T cells and M1-type macrophages, while levels of PD-1 and CTLA-4 and M2-type macrophages tended to decrease after treatment. ICT also inhibits ROS generation, suppresses PI3K/AKT and MEK/ERK/p38 signalling pathways, and inhibits NETs-induced tumor metastasis. Decreased IL-6 expression forms a regulatory feedback loop through the JAK2/STAT3/IL-6 axis. Combining ICT with DNase I reduced the production of NETs promoting tumor invasion and metastasis, while combining ICT with immune checkpoint inhibitors, primarily the PD-1 inhibitor, reduced tumor growth. ICT inhibits lung metastasis by reducing its number and size, inhibits N-cadherin expression, increases E-cadherin expression, inhibits EMT and NETs-enabled tumor stem cell formation (127).

#### 3.5.4 Prostate cancer

##### 3.5.4.1 NETs-related predicting efficacy of anti-cancer therapy

NETs-related signature (NETs) has excellent predictive value in predicting the efficacy of prostate cancer chemotherapy (158).

### 3.6 Gynecological cancers

#### 3.6.1 Ovarian cancer

##### 3.6.1.1 NETs-related predicting efficacy of anti-cancer therapy & nets-related treatment resistance

A high eight-gene signature score of NETs-related genes in ovarian cancer (OC) patients was associated with greater sensitivity to sorafenib and less sensitivity to immunotherapy. In addition, a study of the expression of eight immune checkpoints: LAG3, CTLA4, CD274, PDCD1, PDCD1LG2, TIGIT, showed that they were overregulated in the low-risk group. Also, the estimated IC_50_ values for cisplatin, gemcitabine and veliparib were higher among high-risk individuals (159). In contrast, Wang et al. (160) developed a model with six lncRNAs associated with NETs: GAS5, GBP1P1, LINC00702, LINC01933, LINC02362 and ZNF687-AS1. IC_50_ values for chemotherapeutic drugs (bexarotene, bicalutamide, embelin, GDC0941 and thapsigargin) were higher in patients in the low-risk group. Overall, the high-risk group had less immune cell infiltration and differences in immune checkpoint gene expression compared to the low-risk group, indicating a worse prognosis of the disease in these patients (160). De Amorim et al. (161) found that patients with high-grade serous ovarian cancer (HGSOC) resistant to platinum (PR) were characterized by the presence of a novel deep intron variant, CHEK2, and higher expression of L1, the calprotectin component of NETs. Tamura et al. (162) have demonstrated that NETs capture and inhibit the diffusion of the chemotherapeutic drug doxorubicin (DOX), which may impair its ability to induce apoptosis of ovarian cancer cells. Using 1,000 u/ml of DNase I to degrade NETs increased the diffusion of the drug and enhanced the apoptosis of cancer cells, i.e. improved the response of OC to DOX. The researchers also found that NETs could also trap and inhibit PTX diffusion, but in this case the reduced diffusion was not restored by DNase I. PTX inhibits cell growth by inducing tubulin polymerization and stabilizing it prior to depolymerization, so when bound to polymerized tubulin, it forms large complexes that do not diffuse through micropores, even after using DNase I (162).

##### 3.6.1.2 NETs degradation anti-cancer effects presented on cell lines and mice

Inhibition of NETs formation with GSK484 inhibited tumor progression in OVCAR8-GCSF tumor-bearing mice and significantly delayed the spread of tumor cells to the peritoneum, characteristic of OC. A limitation of the action of PAD4 inhibitors in this case is the lack of a significant anti-tumor effect on ovarian cancer cells without co-occurring neutrophils *in vivo*. The researchers’ results also indicated that pre-treatment of neutrophils with Cl-amidine or DNase 1 significantly inhibited the formation of NETs and consistently reduced the number of ovarian cancer cells attached to them, while it did not decrease the number of cancer cells carried by NETs. Another strategy proposed by the researchers to inhibit NETs is a combination of DNase 1, blockade of granulocyte colony-stimulating factor (G-CSF), which stimulates NETs formation, by an anti-GCSF antibody or its receptor, and removal of neutrophils by an anti-Gr1 antibody. Blockade of NETs was provided by the anti-GCSF antibody in *in vitro* studies, whereas G-CSF itself was not blocked in *in vivo* studies (163).

##### 3.6.1.3 NETs degradation anti-cancer effects presented on mice

CI-amidine and GSK484, reduced net colonisation enabled by NETs, a common site of ovarian cancer metastasis. The number of tumor cells in the peritoneal fluid of mice treated with GSK484 was reduced compared to mice treated with saline solution, and ascites occurring at an advanced stage of disease was also reduced in mice treated with the PAD4 inhibitor. Treatment of mice with DNase also significantly reduced tumor cell implantation in the omentum (164). Singel et al. (165) demonstrated that neutrophils exposed to supernatants of ascites collected from ovarian cancer patients resulted in NETs formation and NE release. A reduction in NE release occurred after heat inactivation and after DNase I administration, also to remove genomic DNA (gDNA) and mitochondrial DNA (mtDNA) (165).

#### 3.6.2 Cervical cancer

##### 3.6.2.1 NETs degradation anti-cancer effects presented on mice

Ning et al. (42) showed that DNase 1 and chloroquine are effective in inhibiting lymph node metastasis occurring with cervical cancer induced by NETs. The mechanism of action of chloroquine, an antimalarial drug, is inhibition of Toll-like receptors (TLRs). Inhibition of TLRs, specifically TLR2, prevents interaction with NETs and thus inhibits activation of the P38-MAPK/ERK/NFκB pathway, which increased the migratory capacity of cervical cancer cells. The drug has also been described to alleviate the hypercoagulation associated with NETs (42).

#### 3.6.3 Breast cancer

##### 3.6.3.1 NETs-related predicting efficacy of anti-cancer therapy & nets-related treatment resistance

In a study by Jiang et al. (166) response to chemotherapy and immunotherapy was associated with the expression of NETs-related lncRNAs. Huang et al. (167) identified five NETs-related genes and constructed subgroups based on this, with patients with triple-negative breast cancer (TNBC) of the high-risk group having a less favorable response to therapy compared to patients with TNBC of the low-risk group. The low-risk patient group instead was enriched in the Wnt signaling pathway, and its inhibitors (Wnt-C59, IWP-2 and XVA-939) had higher sensitivity in patients in this group, as confirmed by *in vitro* studies. In addition, low-risk patients with TNBC treated with radiotherapy had a better therapeutic response. The IC_50_ values of chemotherapy drugs (cisplatin, gemcitabine, olaparib, thalazoparib and vincristine) in high-risk breast cancer (BC) patients were higher than in the low-risk group (167). However, according to a study by Mousset et al. (168) cisplatin- or adriamycin/cyclophosphamide-related chemotherapy used to treat breast cancer metastasis to the lungs induced NLRP3-associated IL-1β secretion by tumor cells, which induced neutrophil recruitment and NETs formation, resulting in a reduced response to therapy in the mice tested. Resistance to chemotherapy in this case is associated with two proteins also associated with NETs: integrin-αvβ1, which captures latent Transforming Growth Factor β (TGF-β), and MMP-9, which cleaves and activates trapped latent TGF-β. Through TGF-β activation, tumor cells undergo EMT, which correlates with resistance to chemotherapy. Treatment with a PAD4 inhibitor or DNase I overcame neutrophil-dependent chemoresistance, but had no effect on the number of tumor cells in mice not given chemotherapy, while *in vitro* inhibition of PAD4 improved the efficacy of chemotherapy. IL-1β blocking antibody inhibited the formation of NETs, reduced neutrophil recruitment and improved the response to chemotherapy. In combination with a PAD4 inhibitor, short-term IL-1β inhibition led only to a statistically insignificant reduction in lung neutrophil recruitment, excluding neutrophils evoked by tumor cells. Long-term PAD4 inhibition reduced IL-1β levels induced by chemotherapy in metastatic lungs (168). Wei et al. (169) discovered that pretreatment with GSK484 enhanced the irradiation-induced (IR) inhibitory effects on TNBC cell proliferation, migration and invasion, and facilitated their apoptosis, which was tested on two TNBC cell lines: MDA-MB-231 and BT-549. *In vivo* studies showed that combined treatment with IR and GSK484 showed a marked decrease in tumor growth in contrast to treatment with IR alone or GSK484 alone (169).

##### 3.6.3.2 NETs degradation anti-cancer effects presented on cell lines

In a study by Safarulla et al. (170) blocking the formation of NETs using Sivelestat, significantly reduced the influx of neutrophils towards metastatic BC cells, but not to their parent tumor. NE inhibition blocked the ability of neutrophils to stimulate invasion of human BC cells, as did NADPH oxidase inhibition. Nawa et al. (171) demonstrated that the combined use of Sivelestat and trastuzumab may be a therapeutic strategy for HER2-positive BC due to NE inhibition, which enables tumor growth *via* tumor growth factor-α (TGF-α), which in turn blocks HER2 down-regulation enabled by trastuzumab. NE enhances cell growth with phosphorylation of EGFR, HER2 and ERK1/2 in BC cells (171). Removal of PAD4 from BC cells (4T1) reduced the rate of tumor growth in a model and reduced their metastasis to the lungs. DNase I treatment also reduced lung metastasis in PAD4-positive as well as PAD4-negative cells, but did not change the number of CTCs (172). A study by Martins-Cardoso et al. (173) on BC cell lines showed that DNase-mediated digestion of NETs had little effect on tumor cell migration, as well as on CXCL8 and MMP9 gene expression. In another study by Martins-Cardoso et al. (174) NEi was found to reduce the expression of metastasis-related genes. In MCF7 cells, the inhibitor reduced the effect of NETs on the expression of CD44, IL-6 and F3 genes, but not ZEB1 and CXCL8, whereas in T-42D cells, it disrupted the expression of all the mentioned genes except ZEB1 (174). Zhao et al. (175), found that DHT, a bioactive compound in *Salvia miltiorrhiza Bunge* (*S. miltiorrhiza*), blocked NETs formation by reducing TIMP1 expression. Researchers initially investigated the effects of four tanshinones (DHT, tanshinone I (Tan I), tanshinone IIA (Tan IIA), and cryptotanshinone (CPT)) on different breast cancer cell types, where DHT showed the most significant inhibitory effect. In studies conducted, DHT inhibited the growth of BC cells more strongly than breast epithelial cells, also inhibited the healing, invasion and migration of BC cells and blocked the progression and spread of BC metastases in lung tissue (175). Cholesterol biosynthesis induced by ASPP2 depletion in BC cells promoted NETs formation *in vitro* and in lung metastases in mice intravenously injected with ASPP2-deficient breast cancer cells. ASPP2, a tumor suppressor and activator of p53, inhibits 3-hydroxy-3-methylglutaryl-CoAreductase (HMGCR) expression. Cholesterol synthesis inhibitors, simvastatin (Simvastatin), which is also an HMGCR inhibitor, and berberine (BBR), effectively blocked NETs formation induced by ASPP2 depletion. DNase I administration inhibited the invasion of ASPP2-depleted cancer cells, indicating that NETs are involved in the process. Also, the expression of Coiled-coil domain containing protein 25 (CCDC25) and caveolin-1, increased in lung metastases from ASPP2-depleted mice, was attenuated by treatment with cholesterol biosynthesis inhibitors or DNase I. The lipid rafts inhibitor piceatannol also reduced CCDC25 expression. Given the proven involvement of NETs in BC metastasis, targeting cholesterol biosynthesis may be a promising therapeutic strategy for their treatment (176).

##### 3.6.3.3 NETs degradation anti-cancer effects presented on mice

DNase I and GSK484 treatment significantly reduced the number of micrometastases in the lungs 24 hours after intravenous injection of labeled tumor cells (13). NETs stimulated invasion and migration of BC cells *in vitro*, and inhibition of this process with DNase I abolished pro-neoplastic targeting of cells. Treatment with DNase I-coated nanoparticles, where the nanoparticles were thought to increase the stability of the enzyme, reduced lung metastasis in mice, while primary tumor growth was unaffected (15). Another DNase delivery system was developed by Herre et al. (9), based on an adeno-associated virus (AAV) vector. It consists of a KP1 capsid and an expression cassette encoding a hyperactive mouse DNase I (AAV-mDNase I) under the control of a liver-specific promoter. The aim of using such a vector is to maintain elevated expression and activity of serum mouse DNase I for at least eight months. After the use of AAV-mDNase I, the proportion of mice in which lung metastases could be observed decreased (9). Inhibition of cathepsin G, a protease associated with NETs, blocked the ability of neutrophils to promote invasion without affecting the ability of tumor cells to invade and also reduced the proliferation of NETs (15). Another cathepsin, cathepsin C (CTSC), involved in the formation of neutrophil serine proteases (NSPs, neutrophil serine proteases), the components of NETs, secreted by the tumor promotes BC metastasis to the lungs via NETs, among others (177, 178). Xiao et al. (178) discovered that CTSC activates neutrophil membrane-associated proteinase 3 (PR3), which activates interleukin-1β (IL-1β) and nuclear factor κB activation, thereby increasing IL-6 and CCL3 expression to recruit neutrophils. The resulting axis induces ROS production by neutrophils and the formation of NETs, which degrade thrombospondin-1 (TSP-1) and promote metastatic tumor cell growth in the lung. Administration of the CTSC inhibitor, AZD7986, effectively inhibited breast cancer metastasis to the lung in a mouse model. Inhibition of PR3 with Sivelestat or IL-1β with a neutralising antibody, but not inhibition of NE or CTSG, reversed CTSC-induced p65 phosphorylation and IL-6 and CCL3 expression. Blocking IL-1β secretion in neutrophils with a lysosome inhibitor also led to inhibition of CTSC-induced neutrophil recruitment. Treatment of mice with an IL-1β-neutralising antibody had no clear effect on primary tumor growth, but effectively inhibited CTSC-increased levels of circulating IL-6 and CCL3, as well as lung metastasis in mice. Inhibition of IL-1β, p38 and ROS production also suppressed CTSC-induced NETs formation in the body and lung. Addition of Sivelestat/CI-amidine/DNase I to neutrophils cultured with cancer cells inhibited NETs formation and blocked the effects of CTSCs. Treatment of mice with GSK484, also inhibited lung metastasis and NETs formation induced by breast cancer cells overexpressing CTSCs, with no significant effect on primary tumor growth (178). Sivelestat, NEi, also reduced the proliferation of NETs induced by cancer cells (15). The NADPH oxidase inhibitor, apocynin, inhibited the formation of NETs and inhibited neutrophil-stimulated tumor cell invasion (15). Also, the PAD4 inhibitor, Cl-amidine, reduced NETs formation and blocked the ability of neutrophils to promote invasion (15). In contrast, in a study by Várada et al. (179) chronic use of rhDNase I had no effect on primary breast tumor growth. Zhu et al. (180) demonstrated that the PAD4 inhibitor inhibits NF-κB and NETs formation, which reduces BC growth and metastasis. The essence of the mechanism at work is that NETs promote breast cancer progression and factors that originate from cancer cells, IL-8 and G-CSF, stimulate neutrophils to form NETs. NETs increased the interaction of the NF-κB essential modifier (NEMO) with IκB kinase (IKK)α/β and enhanced NF-κB activation. Peptide NBD, corresponding to the NEMO-binding domain (NBD) as a selective NF-κB inhibitor, interfered with the NETs-dependent interaction of NEMO with IKKα/β and abolished NF-κB activation *in vitro*. NBD peptide also reduced IL-8 levels and NETs formation, as evidenced by decreased levels of MPO-DNA and citH3 complexes in the circulation of NBD peptide-treated mice, and inhibited primary tumor growth and/or lung metastasis in mouse models of BC (180). Yu et al. (181) identified resveratrol (RES), a silent information regulator-1 (SIRT1) agonist, which inhibited NETs formation after CTSC treatment. The action of RES is to inhibit histone H3 citrullination, while the agonist action abolished the specific deficiency of SIRT1 in neutrophils that promoted NETs formation and BC metastasis to the lung. *In vivo*, RES reduced primary tumor volume and significantly impeded BC metastasis to the lung in a mouse model; researchers also observed lower serum levels of MPO-DNA and NE-DNA complexes after treatment and lower levels of MMP-2, MMP-9, E-cadherin and pro-inflammatory cytokines (IL-1β, IL-6 and TNF-α) in the metastatic lung. In contrast, tumor-infiltrating CD8+ T cells increased, and levels of tissue inhibitor of metalloproteinase-1 (TIMP-1), N-cadherin and Snail increased in the metastatic lung. In BC, RES has been shown to affect every stage of tumor transformation as well as inducing cell cycle arrest and apoptosis. Researchers also mention that, in addition to RES, pentoxifylline, cepharanthine, colchicine, artesunate, dihydroartemisinin and piceatannol also show therapeutic potential with a mechanism similar to RES, dependent on citrullinated H3 or NADPH, ROS, elastase, key pyroptosis execution protein (GSDMD), associated with the formation of NETs (181). Kaempferol (kaem) is a flavonoid that has the ability to inhibit both primary BC tumor growth and its metastasis to the lungs in a mouse model. The addition of GSK484, an inhibitor of NETs, completely abolished the inhibitory effect of kaem on metastasis, while having little or no effect on primary tumor growth, indicating the specificity of kaem’s action on NETs. Addition of the ROS scavenger DPI abolished kaem’s effect on NETs, suggesting the involvement of the flavonoid in NADPH/ROS-NETs signalling. Also, the use of DNase I inhibited the pro-proliferative effects of neutrophils and p-p38 and p-AKT signalling, which NETs potentially use for pro-tumorigenic activities (182). Zhu et al. (183) synthesised cationic oligopeptides with specific numbers of arginine (R) and glycine (G), in this case oligoarginines R5, R7 and R9, which inhibited the interaction of CCDC25 with NET-DNA. Consequently, cell migration and metastasis to the liver and lung of tumors in mouse models of TNBC was inhibited (183). Ye et al. (184) developed a startegy to regulate iron metabolism to reduce the formation of NETs, which would be expected to improve the immune response in TME. The researchers developed a peptide-drug conjugate (PDC) based on transformable iron nanochelate (TIN) equipped with the ability to regulate neutrophil iron metabolism. The mechanism of action of TIN is to expose iron-binding motifs through NE-mediated morphological transformation from nanoparticles to β-sheet nanofibres, which further evolve into stable α-helix nanofibres upon chelation with iron (II) ions, whose regulation inhibits the formation of NETs. TIN in combination with the PAD4 inhibitor, GSK484, synergistically enhanced anti-PD-L1 treatment, as the efficacy in tumor growth inhibition was as high as 93.3%, as tested in BC mice. The tumor growth inhibition rate in mice treated with TIN + GSK484 increased to 87.5%. Tumors from mice treated with TIN + GSK484 had 1.8-fold higher levels of T cells and a 2.2-fold increase in the production of IFN-γ from T cells in tumors, which was also observed in mice treated with TIN + aPD-L1, indicating an increased anti-tumor response compared with control mice (184). TGF-β is necessary in promoting BC recurrence after surgery, which is mediated by NETs. The principle of NETs formation has been used to construct a surgical hydrogel. The hydrogel is prepared based on the electrostatic interaction between histidine (His) and sodium alginate (Alg). The electrical properties of His in the hydrogel lead to the local release of anti-TGF-β. The hydrogel system is a beneficial therapeutic agent due to its ability to specifically and selectively release the drug at the target site, in this case the site of NETs formation. The group that was treated with hydrogel showed better efficacy in reducing metastatic lesions compared to the group in which hydrogel was not used. Hydrogel can mimic the process of NETs formation, release drugs and use the principle of NETs formation to block the mechanisms of recurrence promoted by NETs (185). Lu et al. (186) formulated a micellar nanoparticle of low-molecular-weight heparin and astaxanthin (LMWH-AST/DOX, LA/DOX NP) loaded with DOX to inhibit BC metastasis to the lung and liver. Its mechanism is to inhibit NETs formation, reduce neutrophil recruitment and MPO expression in the liver and MDSCs in the lung and tumor by blocking P-selectin, inhibiting NF-κB and STAT3 signalling pathways. In the tumor itself, the molecule has the ability to reduce ROS, interleukin 10 (IL-10) and nitric oxide (NO) levels (186).

##### 3.6.3.4 NETs degradation anti-cancer effects presented on cell lines and mice

Kong et al. (187) based on their findings about the effect of NETs on the formation of cancer metastasis through a self-reinforcing feedback loop involving two steps: hypoxia-induced aerobic respiration of mitochondria promotes the formation of NETs, which in turn enhance mitochondrial metabolism to exacerbate the hypoxia often present in TME, developed two strategies to nullify NETs. The first is a nanoparticle with DNase I and 5-hydroxytryptamine (5-HT) on the surface to specifically recognise MPO (5HT-NP@D), while the second is a mitochondria-targeting polymer consisting of a water-soluble N-(2-hydroxypropyl)methacrylamide copolymer backbone (HPMA) that was conjugated to the hydrophobic cytotoxic drug camptothecin (CPT) and a mitochondria-targeting peptide (RLA) on the side chains (p-TC-RLA). The function of the nanoparticle is to eliminate NETs and inhibit mitochondrial biogenesis induced by them, while the function of the polymer is to damage mitochondria and alleviate non-oxidation, i.e. synergistically, nanoparticles and polymers completely interrupt the presented feedback loop between NETs and mitochondria. In TNBC tumor-bearing mice, combination therapy effectively inhibited tumor growth compared to monotherapy, with an overall tumor growth inhibition rate of 55.5%; at the highest drug dose, the anti-tumor effect was 70%, and anti-metastatic effects were also observed (187). In a study by Yang et al. (188) inhibition of NETs formation and inhibition of liver metastasis were observed after administration of DNase I to breast tumor mice. DNase I administration also abolished the promotion of migration and adhesion of MDA-MB-231 breast cancer cells by the NET-DNA complex. Also, pretreatment of isolated NET-DNA with DNase I abolished the interaction between CCDC25 and NET-DNA, which enabled migration, adhesion and proliferation of tumor cells. *In vitro*, the CCDC25 antibody inhibited NETs-induced tumor cell migration, adhesion and cytoskeletal remodelling and inhibited liver metastasis when MDA-MB-231 cells were injected into mouse spleens (188).

##### 3.6.3.5 NETs degradation anti-cancer effects presented on mice and human models

Nanoparticulate poly (aspartic acid) derivatives (cANPs), due to their strong affinity for DNA and retention in the liver, reduce levels of NET-DNA hepatic infiltration, leading to significant inhibition of breast cancer tumor metastasis in mice and in human metastatic models of BC and CRC (189). Liang et al. (189) in an attempt to disrupt the interaction between NET-DNA and CCDC25, used poly (aspartic acid)-based cationic materials that inhibit NET-DNA-dependent chemotaxis and tumor cell migration through its electrostatic binding.

### 3.7 Hematological cancers

#### 3.7.1 Multiple myeloma

##### 3.7.1.1 NETs-related treatment resistance

In multiple myeloma (MM), NETs through DNA absorb anthracyclines, preventing their anti-tumor activity and reducing their efficacy (190). Lin et al. (190) observed that the presence of purified NETs protected human cancer cells from doxorubicin-induced apoptosis, a mechanism abrogated by DNase. A similar effect was not observed after the use of PTX. Interestingly, DNase administration alone did not result in an anti-tumor effect, while myeloma symptoms did not appear in MM mice that received the combination of doxorubicin and DNase. The PAD4 inhibitor, BMS-P5, showed a moderate anti-tumor effect on MM, while in combination with doxorubicin, it exhibited potent anti-tumor activity characterized by prolonged survival (190).

##### 3.7.1.2 NETs degradation anti-cancer effects presented on cell lines and mice

Li et al. (191) showed that mouse and human MM cells stimulate histone H3 citrullination and NETs formation. MM cells were unable to induce NETs formation in PAD4-deficient neutrophils. This process is inhibited by pharmacological inhibition of PAD4 with the specific small molecule BMS-P5. Administration of BMS-P5 to mice with MM delayed the onset of symptoms and progression of the disease. The ability of BMS-P5 to inhibit NETs formation was compared with PAD inhibitors: Cl-A inhibitor and GSK-484. All three compounds significantly reduced MM-induced histone H3 citrullination and NETs formation (191).

#### 3.7.2 Diffuse large B-cell lymphoma

##### 3.7.2.1 NETs degradation anti-cancer effects presented on cell lines, mice and human tissues

Higher amounts of NETs in plasma and tumor tissues were associated with poor prognosis in patients with diffuse large B-cell lymphoma (DLBCL). In a study by Nie et al. (53) NETs *in vitro* increase cell proliferation and migration, while *in vivo* they increase tumor growth and lymph node dissemination. DLBCL-derived IL-8 interacted with its receptor (CXCR2) on neutrophils, resulting in the formation of NETs, which directly increased the expression of Toll-like receptor 9 (TLR9) pathways in DLBCL and subsequently activated the NFκB, STAT3 and p38 pathways, promoting tumor progression. Disruption of NETs formation, including blocking the IL-8-CXCR2 axis or inhibiting TLR9, can delay tumor progression. The pro-tumorigenic properties of NETs were attenuated after administration of DNase I and an NE inhibitor. Inhibition of CXCR2 *in vivo* also reduced NETs formation and DLBCL progression, just as TLR9 inhibition inhibited growth and lymph node metastasis in DLBCL patients (53).

#### 3.7.3 Acute leukemia

##### 3.7.3.1 NETs-related treatment resistance

Histones released from leukemic cells during the formation of extracellular traps, mainly containing the histone-DNA complex and NE, induce endothelial activation, which may protect leukemic cells from spontaneous and chemotherapy-induced death (192).

#### 3.7.4 Lymphatic leukemia

##### 3.7.4.1 NETs degradation anti-cancer effects presented on mice

Salganik et al. (193) studied the effect of DNase I injection in mice with spontaneous lymphocytic leukaemia. The study showed that DNase I resulted in a reduction in lymph node size and an increase in survival time by 12 weeks (194).

#### 3.7.5 Acute promyelocytic leukemia

##### 3.7.5.1 NETs degradation anti-cancer effects presented on cell lines and human tissues

Ma et al. (195) found that a small percentage of acute promyelocytic leukemia (APL) cells release extracellular DNA traps in untreated patients. Inhibition of autophagy by pharmacological inhibitors or by small interfering RNAs against Atg7 attenuated LC3 autophagy formation and significantly reduced the generation of extracellular traps, which may represent a novel therapeutic pathway (195). NE can promote APL development, and its inhibitor GW311616A inhibited tumor cell growth and induced apoptosis (196).

##### 3.7.5.2 NETs anti-cancer effects presented on cell lines

Li et al. (197) demonstrated that arsenic trioxide (ATO) increased the formation of extracellular traps by acute promyelocytic leukemia (APL) cells through mammalian target of rapamycin (mTOR)-dependent autophagy, which was partially regulated by ROS. In addition, activation of autophagy with rapamycin enhanced the removal of APL leukemia-initiating cells by ATO (197).

##### 3.7.5.3 NETs anti-cancer effects presented on cell lines and mice

Another study showed that the ability of immature neutrophils to release extracellular traps was impaired in APL, while mature neutrophils produced traps associated with activated platelets. In addition, the combination of all-trans-retinoic acid with ATO induced the differentiation of immature neutrophils, and increased the release of traps from mature neutrophils, excessive amounts of which damaged endothelial cells, causing leakage of blood cells. Administration of DNase 1 alleviated endothelial damage and reduced blood cell leakage (198).

#### 3.7.6 Acute myeloid leukemia

##### 3.7.6.1 NETs-related predicting efficacy of anti-cancer therapy

Zhong et al. (199) showed that patients with acute myeloid leukemia (AML) with high expression of NETs-related genes also have elevated expression of immune checkpoint genes: PD-1, PD-L1 and CTLA4. Similarly, patients with a high risk score had a favorable response to anti-PD-1 therapy, that is, they benefited more from immunotherapy. Compared to the low-risk group, a higher percentage of patients in the high-risk group did not respond to chemotherapy. In addition, the low-risk group showed greater sensitivity to GSK-1838705A, while the high-risk group showed greater sensitivity to 17-AAG (tanespimycin), bosutinib, CI-1040, dowitinib, foretinib, crenolanib, linifanib, selumetinib and trametinib (199).

##### 3.7.6.2 NETs anti-cancer effects presented on cell lines

*In vitro* co-culture of primary AML cells with NETs inhibited the growth of AML cells, reduced their proliferation and induced apoptosis. Both DNase and heparin abolished the effects of NETs on AML cell proliferation and apoptosis (151). Leukemic cells can form extracellular traps containing leukemia-associated antigens, such as mutant nucleophosmin (NPMc+), which is part of NETs.

##### 3.7.6.3 NETs anti-cancer effects presented on mice

The interaction of NETs with dendritic cells (DCs) enables their activation and maturation toward presentation of antigens caught in the network (197). NETs could therefore serve as carriers for DC-based vaccines (200). Tripodo et al. (200) created a vaccine using DCs loaded with NPMc+ and NETs (NPMc+ NET/DC). It reduced myeloproliferation in mice, promoting the development of antibodies to mutant NPMc and induction of CD8+ T-cell responses (86, 200). In mixed bone marrow chimeras, vaccination impaired NPMc+ expansion and allowed control of aggressive leukemia transduced with mutant NPMc, effectively inducing an anti-leukemic CD8 memory T cell response (200).

### 3.8 Skin cancers

#### 3.8.1 Melanoma

##### 3.8.1.1 NETs degradation anti-cancer effects presented on cell lines and mice

Ivermectin (IVM), used as an antiparasitic drug *in vivo*, inhibits melanoma metastasis to the lungs without affecting tumor growth. IVM significantly inhibited NETs formation after cathepsin B (CTSB) treatment. Tumor-infiltrating MDSCs were significantly inhibited, while the number of CD8+ T cells infiltrating the tumor in the lungs increased after IVM treatment in a mouse model of melanoma. IVM targeted GSDMD, whose direct interaction with IVM significantly inhibited GSDMD oligomerisation, which is required for NETs formation. *In vitro* treatment of CTSB in neutrophils located in the bone marrow significantly promotes NETs formation, which was inhibited by IVM. IVM decreases TGF-β, vascular endothelial growth factor (VEGF) and MMP-9 levels, inhibits gasdermin-dependent pore formation and inhibits thermal swelling of cells, which limits the formation of CTSB-induced reticular structures leading to melanoma metastasis (201). CAFs present in the TME and normally acting pro-tumor, have the ability to induce NETs, which in turn are driven by a ROS-dependent pathway dependent on CAF-derived amyloid β. Inhibition of NETs formation in mouse tumors tilts neutrophils towards an anti-tumor phenotype, preventing tumor growth, and at the same time the enhancement of CAF activation by NETs is blocked. Treatment of melanoma mice with GSK484 and Cl-amidine completely inhibited tumor growth compared to controls, whereas this result was not repeated in a pancreatic cancer model (202).

#### 3.8.2 Malignant melanoma

##### 3.8.2.1 NETs anti-cancer effects presented on cell lines

Schedel et al. (203) found that co-culture of NETs with melanoma cells had a cytotoxic effect on ulcerative melanoma cells, causing necrosis. In *in vitro* studies, melanoma cells attached to NETs through integrin-dependent adhesion. In this cancer, NETs inhibited cancer cell migration. Interestingly, addition of DNase I reversed the inhibitory effect of NETs (203).

### 3.9 Osteogenic tumors

#### 3.9.1 Osteosarcoma

##### 3.9.1.1 NETs-related predicting efficacy of anti-cancer therapy

The high amount of NETs arising in initial diagnostic biopsies in patients with suspected osteosarcoma has been associated with poor response to neoadjuvant chemotherapy. Response to chemotherapy was determined by the percentage of tissue necrosis at the time of definitive surgery (Sazer-Kuntschik score). NETs and only NETs, among other parameters: neutrophil-to-lymphocyte ratio, number of neutrophils infiltrating the tumor, CD3+ T cells or CD8+ T cells, were correlated with the extent of necrosis after neoadjuvant therapy (204).

## 4 NETs-releated complication co-occurring with cancer or related to cancer treatment and ways to limit them

**Figure 4:**
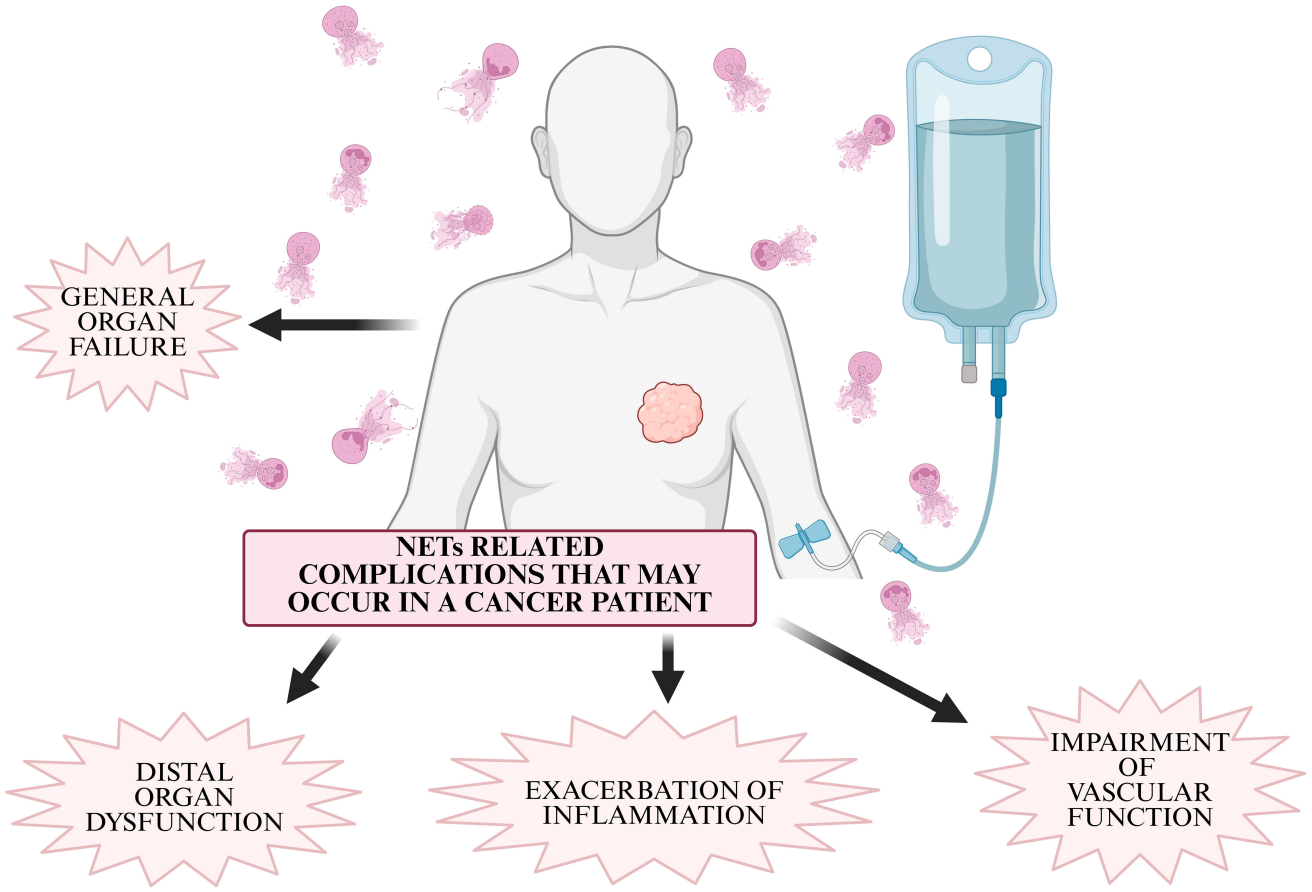
Complications of cancer and its treatment associated with NETs.

Cancer causes a number of side effects in patients, including general organ failure, dysfunction of distal organs, impairment of their vascular function and increased inflammation. It has been shown that these processes may be mediated by NETs, presented in **Figure 4** (205).

Cedervall et al. (205) observed the accumulation of NETs in the vasculature of tumor-bearing mice, which was associated with the up-regulation of pro-inflammatory molecules ICAM-1, VCAM-1, E-selectin, IL-1β, IL-6 and CXCL1. Administration of DNase I restored perfusion in the kidneys and heart to levels observed in the control group, i.e., mice without tumors, and prevented vascular leakage in the blood vasculature of these organs (205). Neutrophil Gelatinase-Associated Lipocalin (NGAL), a biomarker of renal hypoperfusion that is up-regulated in the urine of mice with metastatic BC, was suppressed in mice receiving AAV-mDNase I. This indicates the potential of AAV-mDNase I to reduce cancer-related renal impairment (9).

Cancer-associated thrombosis (CAT) is the second most common cause of mortality in cancer patients and can be intensified by anti-cancer treatment. NETs have been linked to both hypercoagulation, thrombosis, venous thromboembolism (VTE) and CAT directly (206–211). Cao et al. (208) investigated that dunnione (a potent substrate of NAD(P)H quinone oxidoreductase 1, NQO1) attenuates prothrombotic status and pulmonary thrombosis in tumor-bearing mice by inhibiting tissue factor expression and NETs formation. Dunnione increases cellular NAD+ levels in the lung tissues of tumor-bearing mice to restore declining sirtuin 1 (SIRT1) activity, thereby deacetylating NF-κB and preventing tissue factor overexpression in bronchial epithelial and vascular endothelial cells. Dunnione also abrogates the ability of neutrophils to produce NETs by inhibiting histone acetylation and NADPH oxidase activity (208). Chronic treatment with rhDNase I reduced NETs-dependent thrombosis in a mouse model of cancer in a study by Várady et al. (179). The higher mortality rate observed with long-term rhDNase administration was attenuated by treatment with an antibiotic, Ertapenem from the carbapenem group (179). The NETs inhibitor, chloroquine, reduces platelet aggregation, decreases fn tissue factor and reduces hypercoagulation in mice with PDA tumor and associated hypercoagulable state. Administration of DNase I to mice also reduced platelet aggregation (206). Abdol Razak et al. (212) described that degradation of NETs by DNase I and/or prevention of histone-platelet interaction by heparin may become a potential new treatment option for pancreatic cancer patients due to their high risk of VTE. DNase I also reduced the procoagulant effect of NETs in patients with metastatic BC, prolonged plasma clotting time in stage IV patients, and reduced D-dimer and fibrin production (207). Gomes et al. (213) found that IL-1β modulates the expression of G-CSF, which also affects the formation of NETs, and therefore blockade of IL-1R, the IL-1β receptor, with its inhibitor anakinra abolishes the prothrombotic state observed in breast tumor-bearing mice. Researchers have also shown that DNase I and GSK484 have the ability to attenuate clot formation in mice with breast tumor (213). Treatment with recombinant human DNase 1 reversed the prothrombotic phenotype of breast tumor mice, confirming the involvement of NETs in this pathology (214). Compensation of DNase I downregulation, associated with reduced NETs formation in bladder cancer TME, reduces the risk of CAT (25). Also in gastric cancer patients, NETs increased the potential of plasma to generate thrombin and fibrin, an effect that was reduced by DNase administration (215). In another study, a combination of DNase I, activated protein C and Sivelestat markedly abolished the procoagulant activity of NETs in gastric cancer patient samples by simultaneously inhibiting NETs, phosphatidylserine and P-selectin activity on platelets (216). The promyelocyte extracellular chromatin released during APL increases the generation of thrombin and plasmin, shortens the plasma clotting time of APL cells and increases fibrin formation, while this effect was inhibited by DNase I. Extracellular chromatin is cytotoxic to endothelial cells, and together with phosphatidylserine on APL cells provide platforms for fibrin deposition and make clots more resistant to fibrinolysis (217). The predisposition to hypercoagulation caused by NETs in patients with oral squamous cell carcinoma (OSCC) was attenuated by the use of DNase I (218). The ascites that occurs in women with ovarian cancer is also prothrombogenic, Singel et al. (165) found that protease inhibitors were slightly more effective at preventing platelet activation compared to DNase I, suggesting that there are multiple pathways for platelet activation in cancer patients, not always associated with NETs. Wolach et al. (219) showed that neutrophils from patients with myeloproliferative neoplasms (MPNs) form NETs, which has been linked to thrombosis. Also, mice with the most common molecular driver of MPNs, *Jak2^V617F^*, have an increased propensity to form NETs and thrombosis. Inhibition of JAK-STAT signaling with the clinically available JAK2 inhibitor, ruxolitinib, abolished NETs formation and reduced thrombosis in a mouse model of deep vein stenosis. Moreover, expression of PAD4, which is required for NETs formation, is increased in *JAK2^V617F^*-expressing neutrophils and that PAD4 is required for thrombosis formation *in vivo* (219). Diosmetin reduced NETs formation by decreasing ROS, which reduced inferior vena cava thrombosis in an animal model of thrombosis, indicating a potential application in CAT (220). In contrast, although pancreatic cancer cells and pancreatic cancer cell-induced platelets induce the formation of NETs, which promote clot formation when exposed *ex vivo*, after pretreatment with DNase I, platelets continued to adhere and spread to NETs, albeit to a lesser extent. The results of this study suggest that the protein component of NETs is also capable of promoting platelet activation and adhesion (212). Treatment of melanoma mice with GSK484, but not Cl-amidine, reduced von Willebrand coagulation factor (vWF) levels, while fibrinogen levels remained unchanged, suggesting a reduction in NETs-induced thrombosis (202).

Cardiovascular disorders commonly occur in cancer patients, and there is a separate category of disorders: cancer treatment-related cardiovascular toxicity (CTRCT) (221). The association of such disorders with NETs in breast cancer patients was investigated by Zeng et al. (222). In their study, the use of DNase 1 partially reversed changes in the levels of myocardial enzymes, lactate dehydrogenase (LDH) and malondialdehyde (MDA), reduced the distribution of NETs, blocked increasing Bax expression and decreasing Bcl-2 levels in breast cancer liver metastasis tissues studied. The researchers also examined cardiac muscle, whose damage worsened as the metastasis progressed, and administration of DNase 1 may reduce the severity of damage (222).

Not only the cancer itself, but also its treatment, most notably chemotherapy, causes a number of side effects, some of which have been linked to NETs. Such side effects include intestinal damage, caused by irinotecan hydrochloride (CPT-11) used, for example, in advanced colorectal cancer (223). Bai et al. (223) investigated the potential mechanisms of action of phenethyl isothiocyanate (PEITC), an isothiocyanate found in cruciferous (cabbage) plants, in inhibiting NETs and ameliorating chemotherapeutic intestinal injury, in which there is increased neutrophil activation, production of NETs that damage the intestinal epithelium, ischemia and increased expression of inflammatory factors. In a study in chemotherapy-treated mice, PEITC prolonged clotting time, improved intestinal microcirculation, inhibited the expression of inflammatory factors, protected intestinal epithelial junctions, and directly inhibited intestinal cell damage (223). A common and serious complication of cisplatin administration, used for example in breast cancer, is acute kidney damage (168). Mousset et al. (168) detected NETs in the kidney of mice with and without tumor after cisplatin treatment, and that inhibition of NETs formation by a PAD4 inhibitor or IL-1β blockade reduced kidney damage. Interestingly, cisplatin treatment increased the number of NETs in plasma even in tumor-free mice, which would suggest induction of NETs by cisplatin alone. In addition, the researchers did not detect NETs in other potentially affected organs, namely the spleen and liver, suggesting the specificity of NETs toward kidney damage (168). Abdominal infectious complication (AIC) after gastrectomy associated with gastric cancer, for example, stimulates neutrophils to release NETs in both the peripheral blood and abdominal cavity (224). Xia et al. (224) found that AIC-induced NETs can facilitate gastric cancer metastasis *in vitro/vivo* in a TGF-β-dependent manner. The researchers therefore used the TGF-β inhibitor LY 2157299 as a potential therapy to reduce metastasis without exacerbating other complications (224). Todorova et al. (225) demonstrated that NET levels, assessment of prothrombotic status via the thrombin-antithrombin complex and plasma exosome levels are associated with pre-symptomatic DOX cardiotoxicity after a single dose of chemotherapy in breast cancer. Researchers have also found that the risk of DOX-induced cardiotoxicity in breast cancer is associated with endothelial dysfunction, inflammation and prothrombotic status (226).

## 5 Current clinical trials related to NETs

**Table 1** shows current clinical trials related to NETs-related cancer treatment based on ClinicalTrials.gov accessed on June 24, 2025 (227).

**Table 1:** Current clinical trials related to NETs-related cancer treatment.

Cancer	Research ID	Phase	Name of drug	Sponsor	Study population	NETs connection	Primary objective
Breast Cancer	NCT05056857	Recruiting	Tamoxifen (TAM)	University of Kansas Medical Center, Kanmsas, USA	18 years and older – female	Determining whether an increased number of NETs in the body has a harmful effect on BC	Examination of the effect of long-term tamoxifen (TAM) treatment on excessive NET formation in breast cancer patients
Colon Cancer	NCT06017141	Recruiting	NS	University of Kansas Medical Center, Kansas, USA	18 years and older – male and female	Correlation of peri-operative clinical and anesthetic outcomes to NETs levels	Evaluation of the differential impact of TIVA versus inhaled anesthesia on NETs inflammation and immunosuppression among patients undergoing cancer surgery
NS	NCT03781531	Recruiting	NS	Danderyd Hospital, Stockholm, Sweden	18 years and older – male and female	To investigate the diagnostic potential of inflammatory markers, including NET markers, in detecting occult cancer in patients with VTE	Identification of novel biomarkers to aid in the detection of occult cancer in patients with venous thromboembolism
Triple Negative Breast Cancer	NCT06355037	Recruiting	Dasatinib combined with Quercetin	Fudan University, Shanghai, China	18 years to 70 years – female		NS
NS	NCT06355245	Recruiting	NS	Danderyd Hospital, Stockholm, Sweden	18 years and older – male and female		Identification of multi-analyte blood test that can detect and map occult cancer within a mixed population of patients presenting with serious but unspecific symptoms
Bladder Cancer	NCT06325423	Not yet recruiting	neoadjuvant chemotherapy	Assiut University, Assiut, Egypt	19 years and older – male and female	NETs as a prognostic factor for response to NAC in MIBC	Predicting response to neoadjuvant chemotherapy in muscle-invasive bladder cancer
Sarcoma	NCT06815666	Not yet recruiting	NS	University Health Network, Toronto, Ontario, Canada	18 years and older – male and female	NETs as a biomarker of inflammation	BAL fluid biomarkers in sarcoma
Hepatocellular Carcinoma	NCT05040347	Completed	NS	The Affiliated Hospital of Qingdao University, Qingdao, Shandong, China	18 years to 80 years – male and female	NETs as a biomarker to predict portal vein tumor thrombosis in patients with hepatocellular carcinoma	The aim of this study was to investigate whether NETs markers can enhance predict portal vein tumor thrombosis in patients with live cirrhosis, so as to establish a novel predictor to guide clinical decision-making
Myeloproliferative Neoplasms	NCT04177576	Completed	NS	University Hospital, Bordeaux, France	18 years and older – male and female	NETs as a biomarker of thrombosis in myeloproliferative neoplasms	Evaluation of new biomarkers of thrombosis in myeloproliferative neoplasms
Solid and Hematological Malignancies	NCT01533779	Unknown status	NS	Tel-Aviv Sourasky Medical Center, Tel-Aviv, Israel	up to 21 years – male and female	The formation of NETs against cancer cell lines and their ability to kill cancer cells	NETs formation following chemotherapy and their role in antitumor activity
Solid Cancers	NCT04294589	Unknown status	NS	Assistance Publique - Hôpitaux de Paris, Boulogne-Billancourt, Haut de Seine, France	18 years and older – male and female	NETs as a biomarker correlated with the occurrence of Venous Thromboembolic Events	Evaluation of NETs in patients with solid cancers associated with a high risk of venous thromboembolic events

## 6 Conclusions

Despite the existence of an increasing number of therapeutic strategies with their treatment, cancers are, and as projections indicate will continue to be, a condition with an increasing number of patients. In order to create an appropriate therapeutic strategy, it is necessary to understand the mechanisms involved in the cancer process, in which it has been proven that NETs may play a key role. Numerous studies have shown that the gene signature associated with NETs allows for the determination of the sensitivity of various cancers to treatment methods used, which enables the selection of appropriate therapy. Moreover, as studies to date indicate, in a significant number of cancers, the degradation of NETs has a positive effect on treatment, which confirms their predominantly pro-tumor nature. It is also important to consider that in some types of cancer, NETs have both pro- and anti-cancer effects. It should be noted that in order to obtain therapeutic benefits, it is necessary to understand all the mechanisms involved in carcinogenesis, and those related to NETs needs further research, for example, the issue of NETs’ involvement in the early and late stages of the disease and potential biomarker validation for patient stratification. The importance and intensive research required by issues related to the predictive value of NETs and the possibilities of their regulation is demonstrated by the number of centers worldwide addressing these issues.
